# Sex-specific associations between socioeconomic status and ideal cardiovascular health among Korean adults: The Korea National Health and Nutrition Examination Survey, 2007–2017

**DOI:** 10.1371/journal.pone.0307040

**Published:** 2024-08-15

**Authors:** Yiyi Yang, Hokyou Lee, Kokoro Shirai, Keyang Liu, Hiroyasu Iso, Hyeon Chang Kim

**Affiliations:** 1 Department of Public Health, Yonsei University Graduate School, Seoul, South Korea; 2 Division of Public Health, Department of Social Medicine, Graduate School of Medicine Osaka University, Osaka, Japan; 3 Department of Preventive Medicine, Yonsei University College of Medicine, Seoul, South Korea; 4 Institute for Global Health Policy Research (iGHP), Bureau of International Health Cooperation, National Center for Global Health and Medicine, Tokyo, Japan; 5 Institute for Innovation in Digital Healthcare, Yonsei University, Seoul, South Korea; Edith Cowan University, AUSTRALIA

## Abstract

Socioeconomic status (SES) has a considerable impact on cardiovascular health (CVH), which may differ by sex. We aimed to investigate sex-specific socioeconomic disparities in CVH among 31,141 individuals aged 25–64 years who participated in the cross-sectional 2007–2017 Korea National Health and Nutrition Survey (KNHANES) and the Life’s Simple 7 metrics were used to define ideal CVH. Latent class analysis was used to estimate overall SES patterns. Logistic regression models were used to estimate sex-specific odds ratios (ORs) and 95% confidence interval (95% CI) for the likelihood of ideal CVH across SES classes, with the highest SES as the reference group. Four SES classes were identified: (1) low class with low education and material property (2.4%), (2) lower-medium class (10.1%) and (3) higher-medium class (43.7%) with increasing material affluence, and (4) high class with highest education and income (43.8%). Lower SES was associated with decreased ideal CVH among women; compared to their high SES counterparts, women with lowest SES were least likely to achieve ideal overall CVH (OR: 0.55, 95%CI: 0.43–0.71). Similar SES gradients in ideal overall CVH for men were also observed but it was less clear (OR (95%CI) for lowest SES: 0.83, 0.51–1.34). Low SES was associated with poorer achievement of ideal CVH with some sex-heterogeneities. Interventions that equalize the distribution of power and resources and targeted sex-specific approaches to empower low socioeconomic subgroups are warranted to prevent the transition from ideal to suboptimal cardiovascular health and to close socioeconomic disparities in CVH among Korean adults.

## Introduction

Socioeconomic status (SES) represents individual’s combined economic and social position determined by a series of indicators, such as education and income. The World Health Organization demonstrates that socioeconomic disproportion result in differential exposure to various determinants of health (i.e., material circumstances, behaviors and biological factors, psychosocial stressors, and healthcare access) in individuals, thus engender differential susceptibility to downstream genesis and progression of illness (S1 Fig in S1 File) [1]. Cardiovascular disease (CVD) typically represents a downstream consequence of a network of genetic heredity, socioeconomic, environmental, and traditional cardiovascular risk factors (e.g., increased blood pressure), among which SES exerts its effect at environment, childhood, and adulthood level throughout one’s life course [2–4]. Low SES in adolescence and adulthood, as well as environmental deprivation, could predispose individuals to an unfavorable risk factor profile that contributes to CVD events in later life. In recent decades, despite reductions in CVD burden has gained through the global effort to treat cardiovascular risk factors through primary and secondary prevention, CVD persists the leading cause of death and a major cause of disability worldwide and SES disparities in CVD have enlarged [5]. Researchers therefore highlight primordial prevention, which targets preventing deteriorating levels of risk factors preceding the occurrence of the preclinical phase of CVD or preventing one’s health status shift from ideal into suboptimal. Suboptimal health status (SHS) is a reversible stage between ideal health and irreversible diseases, characterized by ambiguous health complaints, general weakness, chronic fatigue, and low energy levels, which does not meet any criteria of diseases but lays the seed for future non-communicable diseases including CVD [6, 7]. The pathway from ideal health to suboptimal health then to CVD underscores new solutions for CVD prevention. The American Heart Association (AHA) has introduced the concept of ideal cardiovascular health (ideal CVH) based on seven metrics (Life’s Simple 7 metrics) [8] to emphasize the primordial strategies that prevent the adoption of risk factors and mitigate the progression from ideal health towards suboptimal health and eventually CVD. Ideal CVH was associated with lower odds of suboptimal health [9], lower risk and mortality of CVD and related health inequalities [10, 11]. Higher SES is reported to facilitate ideal CVH profile [12–20]. However, such evidence is limited among East Asian populations [13, 14], especially Korean adults. In addition, most of the previous studies focused on individual SES indicators in isolation (e.g., education), neglecting the comprehensive and complex nature of SES. Furthermore, socio-demographics may modify SES disparities in ideal CVH; SES disparities were found to be stubborn among women [15–18, 21] compared with men with unclear underlying mechanisms. Therefore, we aimed to use the latent class analysis (LCA), a clustering approach for distinguishing population subgroups based on heterogeneity in SES from different dimensions, to evaluate the holistic SES gradient among general Korean adults and further examine its sex-specific relationship associated with the achievement of ideal CVH.

## Methods

### Study design and sample

This study used data from the Korea National Health and Nutrition Examination Survey (KNHANES) [22], a national representative cross-sectional study that assesses the health and nutritional status of Koreans through health interview, physical examination, and nutritional survey. KNHANES utilized a stratified, multi-stage, clustered sampling method to represent entire non-institutionalized Koreans aged >1 year. We pooled data from 11 consecutive years from 2007 to 2017 owing to the availability of data. We limited study subjects to adults aged 25–64 years who tend to have reached the peak of education, occupational, and income classes. We further excluded pregnant women and subjects with missing information on SES indicators and CVH metrics, yielding 31,141 eligible subjects (S2 Fig in S1 File). Written informed consent was obtained from all participants. The KNHANES de-identifies personal information from the published data; the study was therefore exempted from the corresponding institutional review boards of the research institutes.

### SES components

We used a range of self-reported socioeconomic proxies in childhood, adulthood, and environmental dimension for constructing a latent SES complex based on previous literature (Table 1) [23, 24]. Education reflecting human capital of skills and knowledge is associated with occupational and income potential [25] and income was straightforward linked to material resources, including health insurance, house, and living support [26]. Paternal education represents childhood SES of knowledge and resources that parents may transfer to offspring. Residing in rural areas represents environmental deprivation [24]. Occupation was re-categorized according to the Korean Standard Classification of Occupations [27] definition of non-manual work. People below the poverty threshold are given governmental subsidies through the National Basic Livelihood Security System [28] and Medical Aid [29] for they cannot afford the National Health Insurance premiums.

**Table 1:** Components for constructing SES class gradients.

SES domain& indicators	Variable explanation	Re-categorized dichotomous value
**1. Structural determinants**		
**Educational attainment**	Highest education achievement was reported in “primary school”, “secondary school”, “high school”, or “college or above”. Education variable was then categorized into binary level	College-equivalent or above (yes/no)
**Occupation type**	Measured according to the Korean Standard Classification of Occupations in non-manual job (officers, managers, or professionals), manual workers (office workers, sales, machinery operators, agricultural, forestry, or fishery labors, or physical worker), or economically inactive (unemployed or housewife) and further dichotomized	Non-manual worker (yes/no)
**Equivalized monthly average household income level**	Monthly household income including salaries, property income, pension, and government subsidies was equivalized/divided by the square root of the number of household members. It was further categorized into binary level based on the medium value	≥ medium value of 2-million won (yes/no)
**2. Environmental determinants**		
**Residence area**	Residence including metropolitan cities and suburb areas	Residing in metropolitan cities/rural area
**3. Material circumstances**		
**House ownership**	The situation of owning one’s house or apartment, or having a mortgage on it and was dichotomized	Owning at least 1 house (yes/no)
**4. Social welfare system**		
**Governmental subsidies**	Governmental living support for those who are below the national poverty threshold	Receiving/ not receiving
**Medical Aid**	Basic health care service provided for the lower income residents who cannot afford for regional or occupational health insurance	Receiving/ not receiving
**5. Childhood SES**		
**Paternal educational attainment**	Father’s highest educational attainment was measured as same as individual educational attainment and was further dichotomized	≥ high school (yes/no)

### Cardiovascular health metrics and ideal CVH

Life’s Simple 7 metrics of four behavioral metrics (smoking, physical activity [PA], diet quality, body mass index [BMI] and three biological metrics (systolic and diastolic blood pressure [BP], fasting plasma glucose [FPG], and total cholesterol [TC]) were measured and recorded in poor, intermediate, and ideal status, respectively, according to AHA criteria [8] with revisions appropriate for Asian-Pacific residents (S1 Table in S1 File). Details of metric revision are also outlined (S1 File). Height and weight were measured and BMI was calculated as weight divided by height-squared (kg/m^2^). Systolic and diastolic BP were measured using a standard mercury sphygmomanometer (Baum/ USA). Blood samples were collected after at least 8 hours of fasting and were analyzed within 24 hours of being drawn. Concentrations of serum glucose and cholesterol were measured with an ADIVIA1650 (Siemens/ USA) or a Hitachi Automatic Analyzer 7600 (Hitachi/Japan). Ideal behavioral, biological, and overall CVH were defined as having 3–4 behavioral (i.e., non-smoking, BMI 18.5–23 kg/m2, PA at goal levels, and a diet consistent with current guideline recommendations), 3 biological (i.e., untreated TC <200 mg/dL, untreated BP <120/<80 mm Hg, and untreated FPG < 100 mg/dL), and 5–7 combined metrics at ideal status, respectively [8].

## Statistical analysis

Firstly, we ran LCA to fit models by integrating SES indicators into multiple classes and identified the final model with best model fit indices and good interpretability that indicate mutually exhaustive class membership [30] (S1 File). Characteristics of participants and age-adjusted prevalence of ideal CVH metrics reflecting an underlying population in 2010 South Korea census [31] across latent SES gradients were presented. We then estimated the likelihood of achieving ideal CVH (versus poor and intermediate status) across SES gradients with highest SES as the reference level using logistic regression models. We tested age-adjusted and multivariate-adjusted models for confounders including age (continuous), cohabitation (whether married or live with a partner), Charlson Comorbidity Index (CCI) [32], depression (yes/no), stress (yes/no), engagement in high-risk drinking (yes/no), and regular health checkup (yes/no). Missing values for all covariates were less than 0.3%. We further investigated the relative disparities (relative index of inequality) [33] in ideal CVH by sex and age categories. Sensitivity analyses were performed to assess the robustness of the findings. First, we repeated the analysis by education and income in isolation to test the interchangeability of the SES indicators. Second, to address the potential impact of time trend, we repeated analyses separated by two periods (2007 to 2012 and 2013 to 2017). All statistical analyses were undertaken considering complex survey design of stratification, clustering, and population weights, using SAS 9.4 (SAS Institute, Cary, NC) with the statistical significance level set as two-tailed p value <0.05.

## Results

### Latent SES pattern among Koreans aged 25–64 years

We regraded a four-class SES pattern among total participants as adequate (S1 File). The general SES gradients comprised the following: low class (2.4%) with lower than college-equivalent individual and paternal education, low income, governmental subsidies, and Medicaid support but no house; lower-medium class (10.1%) with relatively higher education and income compared to those in the low class; higher-medium class (43.7%) with higher income compared to the precious class and at least one house; and high class (43.8%) with the highest probabilities of individual and paternal college-equivalent education, higher income, and house ownership (Table 2).

**Table 2:** Class membership probabilities and conditional probabilities of SES indicators for latent SES gradients.

SES domain and indicators	Class 1 (n = 761; 2.4%)	Class 2 (n = 3,131; 10.1%)	Class 3 (n = 13,607; 43.7%)	Class 4 (n = 13,642; 43.8%)
Structural determinants of SES				
Education ≥ college	0.165	0.289	0.077	0.931
Non-manual worker	0.041	0.050	0.022	0.362
Income level ≥ 2 million won	0.012	0.111	0.491	0.681
Environmental SES				
Residence in metropolitan cities	0.777	0.893	0.720	0.906
Material circumstances				
Owning ≥1 house	0.176	0.007	0.848	0.738
Social welfare system				
Not receiving subsidies	0.098	0.941	0.973	0.992
Not receiving Medical Aid	0.318	0.999	0.998	1.000
Childhood SES				
Paternal education ≥ high school	0.166	0.328	0.131	0.569
Description	Lower education, income Manual worker or is unemployed Residing in cities Owning no house Receiving subsidies and Medical Aid Lower paternal education	Lower education, income Residing in cities Manual worker or unemployed Residing in cities Owning no house Not receiving subsidies or Medical Aid Lower paternal education	Lower education Relatively higher income Manual worker or is unemployed Residing in cities Owning a house Not receiving subsidies or Medical Aid Lower paternal education	Higher education and income Manual worker or is unemployed Residing in cities Owning a house Not receiving subsidies or Medical Aid Higher paternal education
Generated SES gradient	Low	Lower-medium	Higher-medium	High

### General characteristics and prevalence of ideal CVH by SES classes

**Fig 1:**
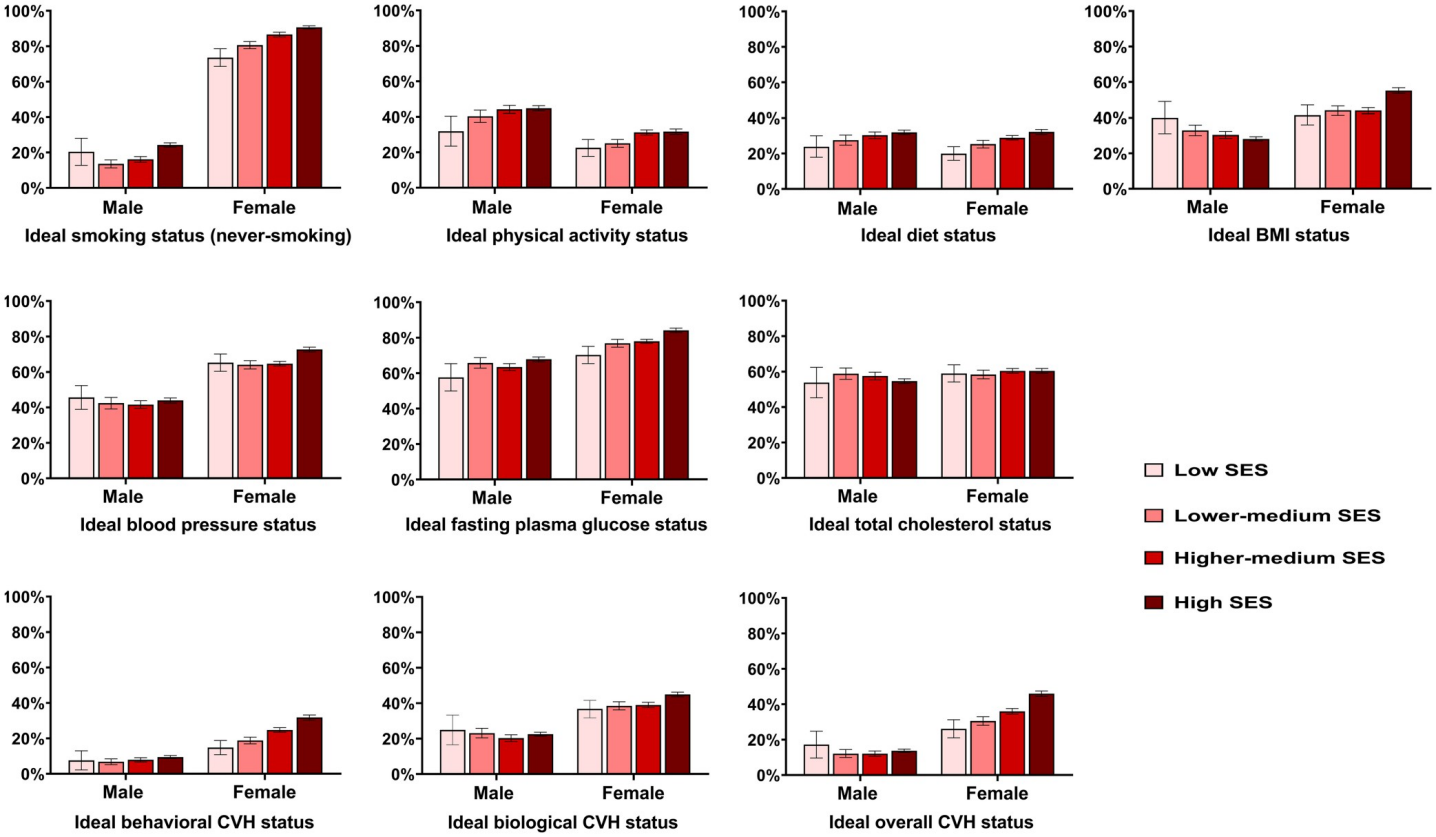
Age-standardized prevalence* of ideal CVH^†^ by sex and SES classes. *Estimates were directly standardized to a population derived from the 2010 Korean census by sex and 10-year age categories. ^†^ Ideal behavioral CVH was defined as having 3–4 out of 4 behavioral metrics (smoking, PA, diet, and BMI) at ideal status. Ideal biological CVH was defined as having 3 biological metrics (blood pressure, fasting plasma glucose, and total cholesterol) at ideal status. Ideal overall CVH was defined as having 5–7 out of 7 total metrics at ideal status.

Table 3 shows that participants belonging to higher SES classes were generally younger, with the exception of the higher-medium class. Regardless of sex, participants with lower SES tend to be more depressed, stressed and they engaged more in high-risk drinking but less in health checkup. Fig 1 presents an upward trend in the age-standardized prevalence of ideal CVH status and its individual components (except for TC) among women correlating with higher SES classes. Similarly, among men, the higher SES was associated with a higher frequency of ideal PA, diet, FPG, and behavioral CVH. Conversely, an ideal BMI was less prevalent among men with higher SES. It was also observed that women achieved ideal CVH more frequently than men, with the exceptions for PA and diet components. It is also notable that less than half of the overall study population attained ideal CVH, particularly in behavioral components.

**Table 3:** General characteristics of KNHANES participants (25–64 years) by SES classes and sex.

	Male (n = 12,590)				Female (n = 18,551)			
	Low (n = 274)	Lower-medium (n = 1,183)	Higher-medium (n = 4,782)	High (n = 6,351)	Low (n = 487)	Lower-medium (n = 1,948)	Higher-medium (n = 8,825)	High (n = 7,291)
Age, y	48.3±0.7	42.5±0.3	49.2±0.2	39.9±0.2	46.5±0.5	41.8±0.3	48.7±0.1	38.2±0.1
Age group								
25–49	48.4 (3.5)	73.6 (1.4)	46.9 (0.9)	80.8 (0.6)	63.8 (2.6)	76.7 (1.1)	49.4 (0.7)	87.4 (81.3)
50–64	51.6 (3.5)	26.4 (1.4)	53.1 (0.9)	19.2 (0.6)	36.2 (2.6)	23.3 (1.1)	50.6 (0.7)	12.6 (0.5)
Cohabit with a partner	46.0 (3.6)	71.6 (1.5)	85.8 (0.7)	70.9 (0.8)	45.0 (2.7)	78.4 (1.1)	88.0 (0.4)	75.4 (0.7)
SES components								
Education ≥ college	17.3 (2.6)	29.4 (1.6)	3.2 (0.3)	96.8 (0.2)	14.0 (1.8)	21.1 (1.1)	1.6 (1.9)	98.9 (0.1)
Non-manual worker	5.0 (1.4)	4.7 (0.7)	2.0 (0.2)	38.3 (0.7)	3.1 (0.8)	2.9 (0.4)	2.0 (0.2)	32.9 (0.6)
Income ≥ medium 2	1.0 (0.7)	0.5 (0.3)	48.1 (0.9)	65.6 (0.8)	0.8 (0.5)	0.4 (0.2)	54.7 (0.7)	66.7 (0.8)
million won								
Reside in cities	74.2 (3.3)	92.6 (1.0)	66.8 (1.4)	90.4 (0.7)	81.1 (2.4)	94.8 (0.7)	75.7 (1.2)	91.5 (0.7)
Owning a house	10.3 (1.9)	0.0 (-)	86.1 (0.7)	71.1 (0.8)	8.4 (1.4)	0.0 (0.0)	86.5 (0.5)	71.9 (0.8)
Government subsidies	95.9 (1.4)	3.9 (0.7)	2.9 (0.3)	1.2 (0.2)	93.2 (1.3)	4.6 (0.6)	3.1 (0.2)	0.9 (0.1)
Recipient								
Medicaid recipient	67.9 (3.4)	0.0 (-)	0.4 (0.1)	0.0 (0.01)	67.7 (2.5)	0.0 (-)	0.1 (0.04)	0.1 (0.04)
Paternal education ≥	14.0 (2.5)	22.4 (1.4)	13.6 (0.6)	55.0 (0.7)	12.4 (1.7)	24.1 (1.2)	16.5 (0.5)	62.1 (0.7)
high school								
Charlson comorbidity	1.1±0.08	0.5±0.03	1.0±0.02	0.4±0.01	0.8±0.05	0.4±0.02	0.9±0.01	0.3±0.01
index								
Having depressive	13.6 (2.4)	1.3 (0.3)	1.7 (0.2)	1.6 (0.2)	17.3 (1.9)	5.9 (0.6)	5.7 (0.3)	3.2 (0.2)
symptoms								
Feeling stress	38.3 (3.4)	30.0 (1.6)	21.2 (0.7)	29.5 (0.6)	46.2 (2.6)	31.7 (1.2)	25.7 (0.6)	30.8 (0.6)
Engaging in high risk	24.1 (2.9)	22.9 (1.3)	25.8 (0.8)	21.4 (0.6)	10.2 (2.0)	7.9 (0.7)	5.0 (0.3)	4.1 (0.3)
drinking								
Regular health-checkup	46.7 (3.5)	52.5 (1.6)	66.8 (0.8)	66.0 (0.7)	51.0 (2.7)	43.5 (1.3)	64.0 (0.6)	59.0 (0.7)

Weighted mean (SE), prevalence±SE were presented wherever appropriate. Missing values of all confounding factors were all <0.3%

**Abbreviations**. CVH, cardiovascular health; SES, socio-economic status; PA, physical activities; BMI, body mass index; BP, blood pressure; FPG, fasting plasma glucose; TC, total cholesterol

### General association of SES with ideal CVH achievement

Table 4 shows the likelihood of ideal CVH associated with SES and other health determinants among total population. A dose-response relationship was found between lower SES and decreased ideal CVH; participants with higher-medium, lower-medium, and low SES had 0.80, 0.66, and 0.64 fold lower potential to achieve ideal overall CVH, respectively, compared to those with high SES. Older participants were 0.97 times less likely to achieve ideal overall CVH. Women were significantly 3.66, 2.60, and 4.40 times more likely to achieve ideal behavioral, biological, and overall CVH, respectively. Participants who were single, divorced or widowed, depressed, stressed, or who engaged in high-risk drinking had poorer achievement of ideal CVH.

**Table 4:** Odds ratios (95%CI) of achieving ideal CVH* associated with socio-demographics, SES gradients, and lifestyle factors among total population.

Item	Ideal overall CVH				Ideal behavioral CVH				Ideal biological CVH			
	Model 1^†^		Model 2^‡^		Model 1		Model 2		Model 1		Model 2	
	OR	(95%CI)	OR	(95%CI)	OR	(95%CI)	OR	(95%CI)	OR	(95%CI)	OR	(95%CI)
Age per 10 year	0.64	(0.62–0.66)	0.72	(0.69–0.76)	1.05	(1.02–1.08)	1.11	(1.05–1.17)	0.44	(0.43–0.45)	0.49	(0.46–0.51)
Age, category												
25–49	1.00		1.00		1.00		1.00		1.00		1.00	
50–64	0.67	(0.60–0.76)	0.97	(0.96–0.97)	1.06	(0.98–1.13)	0.97	(0.84–1.12)	0.20	(0.18–0.21)	0.44	(0.39–0.49)
Sex												
Male	1.00		1.00		1.00		1.00		1.00		1.00	
Female	4.64	(4.32–4.98)	4.40	(4.08–4.75)	3.79	(3.50–4.11)	3.66	(3.36–3.98)	2.87	(2.69–3.06)	2.60	(2.43–2.78)
SES gradient												
High	1.00		1.00		1.00		1.00		1.00		1.00	
Higher-medium	0.76	(0.71–0.83)	0.80	(0.74–0.86)	0.77	(0.71–0.84)	0.80	(0.74–0.87)	0.84	(0.78–0.90)	0.86	(0.80–0.93)
Lower-medium	0.63	(0.55–0.71)	0.66	(0.58–0.74)	0.57	(0.49–0.64)	0.59	(0.52–0.67)	0.88	(0.79–0.97)	0.89	(0.80–0.98)
Low	0.52	(0.41–0.66)	0.64	(0.50–0.81)	0.41	(0.31–0.54)	0.46	(0.35–0.62)	0.81	(0.66–1.00)	0.98	(0.79–1.21)
Cohabitation												
Living with a partner	1.00		1.00				1.00				1.00	
Single, divorced, or widowed	0.84	(0.77–0.91)	0.92	(0.84–1.00)	0.94	(0.85–1.03)	1.01	(0.92–1.11)	0.78	(0.71–0.84)	0.82	(0.75–0.89)
CCI score	0.77	(0.72–0.81)	0.78	(0.73–0.83)	0.92	(0.87–0.98)	0.93	(0.88–0.98)	0.77	(0.72–0.82)	0.79	(0.74–0.83)
Depression												
Yes vs. no	0.68	(0.57–0.81)	0.79	(0.66–0.94)	0.74	(0.62–0.89)	0.85	(0.71–1.03)	0.74	(0.62–0.89)	0.82	(0.68–0.97)
Stress												
Yes vs. no	0.78	(0.73–0.84)	0.82	(0.76–0.88)	0.77	(0.71–0.84)	0.80	(0.74–0.87)	0.86	(0.82–0.95)	0.91	(0.85–0.98)
High-risk drinking												
Yes vs. no	0.46	(0.40–0.52)	0.47	(0.42–0.54)	0.51	(0.44–0.60)	0.54	(0.46–0.63)	0.53	(0.47–0.59)	0.53	(0.47–0.59)
Health checkup												
No vs. yes	0.80	(0.75–0.86)	0.86	(0.80–0.92)	0.80	(0.74–0.86)	0.85	(0.79–0.92)	0.96	(0.89-.1.02)	1.00	(0.94–1.07)

* Ideal behavioral CVH was defined as having 3–4 out of 4 behavioral metrics (smoking, PA, diet, and BMI) at ideal status. Ideal biological CVH was defined as Having 3 biological metrics (blood pressure, fasting plasma glucose, and total cholesterol) at ideal status. Ideal overall CVH was defined as having 5–7 out of 7 total metrics at ideal status

^†^ Model 1 was age-and sex-adjusted for each variable, except for age variables and sex

^‡^ Model 2 adjusted for age, sex and mutually adjusted for all variables

### Sex-specific association of SES and ideal CVH and related disparities

**Fig 2:**
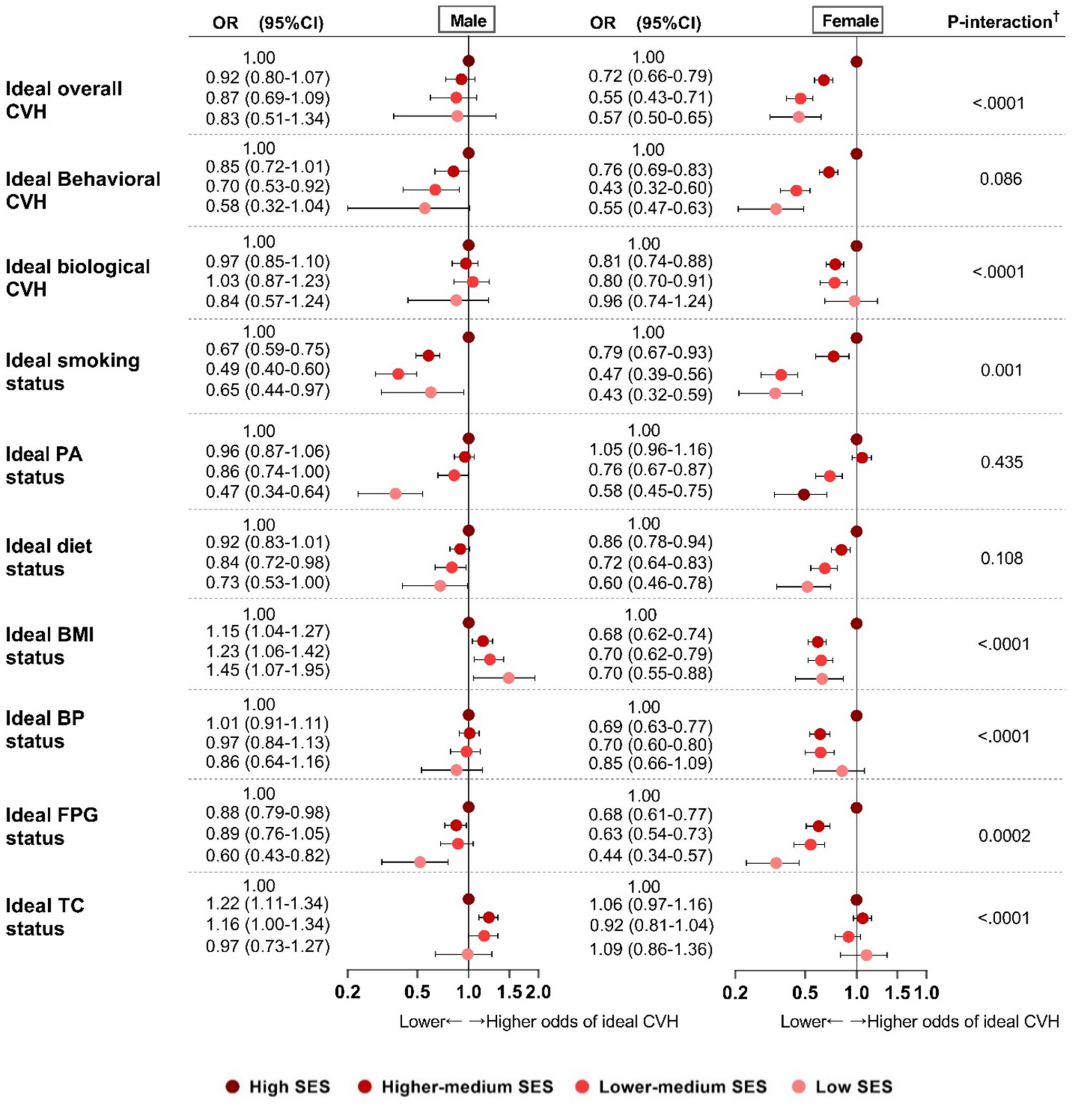
Multivariate-adjusted odds ratios (OR, 95%CI)* of ideal CVH^†^ associated with SES by sex. * Model adjusted for age, cohabitation, CCI score, depressive symptom, stress, high-risk drinking behavior, and health checkup. ^†^ P-interaction tested the interaction effect of SES and sex on each ideal CVH status and metric.

Fig 2 shows a significant and progressive reduction in the likelihood of ideal smoking, PA, diet, FPG metrics, and behavioral and overall CVH associated with a decrease in SES among women. Despite a smaller magnitude, lower SES was also associated with a decrease in ideal PA, diet, FPG, and behavioral CVH among men (all p-trend <0.0001). Sex-different associations between SES and ideal BMI were detected. Women in lower SES classes were associated with worse achievement while their male counterparts were associated with better achievement of ideal BMI, where men with lowest SES were 1.45 (95%CI, 1.07–1.95) times more likely to have an ideal BMI. Table 5 compares relative SES inequalities in ideal CVH by sex and age strata. Generally, SES disparities were higher (i.e., disparities in favor of high SES) for all outcomes except for ideal TC and biological CVH. Compared with women, SES disparities were slightly smaller in men for never smoking, ideal BP, FPG, and behavioral CVH. Moreover, decreased disparities (i.e., disparities in favor of low SES) were observed for ideal BMI and TC among men. Among women, SES disparities in ideal FPG and biological CVH were slightly higher in the younger group than the older group.

**Table 5:** Relative inequalities (RII, 95%CI) of achieving ideal CVH* by sex and age category.

	Total population^†^	By sex^‡^		By age category^§^		By sex and age category^‡^			
		Male	Female	25–49 years	50–64 years	25–49 years male	50–64 years male	25–49 years female	50–64 years female
CVH metrics									
Smoking	1.24 (1.19–1.29)	1.19 (1.14–1.23)	3.50 (2.88–4.26)	1.45 (1.35–1.55)	1.21 (1.09–1.35)	1.22 (1.16–1.28)	1.11 (1.05–1.17)	3.50 (2.86–4.28)	3.92 (1.97–7.80)
PA	1.12 (1.08–1.17)	1.14 (1.06–1.23)	1.11 (1.06–1.16)	1.09 (1.03–1.15)	1.22 (1.14–1.31)	1.07 (0.98–1.18)	1.26 (1.13–1.40)	1.09 (1.03–1.15)	1.20 (1.11–1.30)
Diet	1.12 (1.09–1.16)	1.09 (1.04–1.15)	1.15 (1.11–1.20)	1.11 (1.07–1.15)	1.18 (1.10–1.27)	1.07 (1.01–1.14)	1.18 (1.06–1.31)	1.15 (1.10–1.20)	1.19 (1.08–1.30)
BMI	1.04 (0.99–1.08)	0.90 (0.85–0.95)	1.36 (1.27–1.45)	1.09 (1.03–1.15)	1.07 (1.00–1.14)	0.90 (0.84–0.96)	0.90 (0.83–0.98)	1.39 (1.28–1.52)	1.32 (1.19–1.45)
BP	1.14 (1.08–1.20)	1.07 (1.00–1.14)	1.32 (1.21–1.44)	1.18 (1.08–1.28)	1.16 (1.09–1.24)	1.02 (0.92–1.12)	1.11 (1.03–1.21)	1.63 (1.41–1.89)	1.21 (1.10–1.34)
FPG	1.33 (1.22–1.44)	1.18 (1.07–1.31)	1.77 (1.55–2.03)	1.43 (1.27–1.62)	1.28 (1.15–1.43)	1.15 (0.99–1.35)	1.20 (1.06–1.36)	2.17 (1.79–2.64)	1.42 (1.18–1.70)
TC	0.93 (0.88–0.99)	0.85 (0.78–0.93)	1.01 (0.94–1.09)	0.95 (0.87–1.03)	0.91 (0.84–0.98)	0.85 (0.75–0.96)	0.83 (0.73–0.96)	1.09 (0.96–1.23)	0.96 (0.88–1.06)
CVH									
Behavioral CVH	1.09 (1.06–1.11)	1.04 (1.02–1.07)	1.28 (1.23–1.33)	1.14 (1.11–1.17)	1.16 (1.11–1.20)	1.04 (1.01–1.07)	1.04 (1.00–1.08)	1.28 (1.22–1.33)	1.32 (1.23–1.42)
Biological CVH	1.02 (0.98–1.06)	1.01 (0.97–1.05)	1.03 (0.97–1.09)	1.08 (1.03–1.14)	1.03 (1.00–1.06)	1.02 (0.96–1.08)	1.00 (0.96–1.04)	1.20 (1.10–1.29)	1.06 (1.00–1.11)
Overall CVH	1.06 (1.03–1.09)	1.03 (1.00–1.06)	1.33 (1.26–1.41)	1.19 (1.14–1.24)	1.12 (1.08–1.16)	1.03 (0.99–1.08)	1.02 (0.99–1.06)	1.54 (1.43–1.66)	1.24 (1.16–1.32)

* Ideal behavioral CVH was defined as having 3–4 out of 4 behavioral metrics (smoking, PA, diet, and BMI) at ideal status. Ideal biological CVH was defined as having 3 biological metrics (blood pressure, fasting plasma glucose, and total cholesterol) at ideal status. Ideal overall CVH was defined as having 5–7 out of 7 total metrics at ideal status

^†^ Adjusted for age (continuous) and sex;

^‡^ Adjusted for age (continuous);

^§^ Adjusted for sex

## Discussion

### Main finding

In this cross-sectional study, a gradient pattern among Korean adults was identified in four distinct socioeconomic classes (low, lower-medium, higher-medium, and high). Additionally, lower SES was associated with reduced individual potential of achieving an ideal CVH (especially its behavioral components) and CVH-related SES disparities were more consistent in women than men.

### Regarding previous studies

SES disparities in CVH have been discussed [12–20] and higher SES was uniformly reported to be associated with a favorable CVH profile. A systematic review and meta-analysis [12] concluded that higher educational attainment, employment, higher household income, and low environmental deprivation were all associated with achieving a greater number of ideal CVH metrics. In contrast, low education attainment (less than college) is associated with the poorest achievement of ideal overall CVH (OR: 0.29, 95%CI: 0.19–0.44). This is predictable that low SES predisposes individuals to risk factors that lead to one’s suboptimal CVH state. Studies on East Asians was predominantly on Chinese residents [13, 14, 20] but our study enrich the evidence among Koreans that lower SES was associated with poorer achievement of three aspects of ideal CVH. After adjusting for partnership, comorbidities, depression, stress, high-risk drinking, and health checkup, the association between lower SES and decreased ideal overall CVH were attenuated but remained statistically significant (e.g., multivariate-adjusted vs. age, sex-adjusted OR (95%CI): 0.64 (0.50–0.81) vs. 0.52 (0.41–0.66)). Thus, we presumed that a lower SES deteriorates CVH indeed partially through social support from a cohabited partner [34], psychological stressors, and unfavorable lifestyle.

### Sex- and age-heterogeneities in the association of SES and ideal CVH

Our study further illuminates sex-specific differences. Results pooling 50 studies worldwide [12] showed that overall achievement of ideal CVH metrics was higher for women than for men (except for PA and TC metrics) and similarly, we found a higher prevalence of all ideal CVH metrics (except for PA) in women. Studies in South Korea, US and Europe reported wider SES disparities in smoking [16, 35, 36], PA [16, 37], healthy diet [16, 38], obesity, increased BP and FPG [2, 3, 36, 39], and ideal overall CVH [15–18, 21] in women than men. A Chinese study [14] even reported that high education combining high income was associated with the most ideal CVH metrics in women but not in men (OR (95%CI): 2.59 (1.74–3.87) vs. 1.42 (0.95–2.21)). Consistently, we found that lower SES was associated with decreased ideal CVH and most of its components in a graded manner, except TC metric, among women. For men, this association was also seen, but in less components and with a smaller effect size. The reasons behind magnified SES disparities in ideal CVH remain unclear. One possible explanation is that gender roles influence on socioeconomic constructs that reflect the discrimination and reinforcement of social hierarchies [1]. Given the fact that women tend to be less educated or hired in managing or professional positions and that female workers are less likely to obtain higher income compared to their male counterparts with similar educational or occupational levels, women in higher SES classes may be particularly privileged [2]. Interestingly, we found that low SES was associated with higher achievement of ideal BMI and TC among men. This finding is consistent with studies that reported obesity and elevated lipids to be extra prevalent in men with higher income, in South Korea [3, 21, 36, 39] and other low and middle-to-high income countries (e.g., Congo, Mexico, Thailand, and China) [40–42], whereas higher education and income were associated with decreased obesity and hyperlipidemia for both sexes in developed Western countries (e.g., US and Spain) [2, 16, 18]. This phenomenon reflects the fact that South Korea faced a transition pattern of risk factors epidemiology into developed country. Men with a higher SES in developing countries were more likely to consume high-calorie foods and alcohol and avoid physical activities [40] while healthy diet and physical activity were more prevalent in men with higher SES in high income countries [43], despite that healthy lifestyle was well embraced by women with higher SES in both type of countries [43]. It is also possible that sex-based differences in attitudes toward body image and capacity to control weight modified the sex differences in socioeconomic disparities in ideal BMI. With society’s stronger negative attitude towards obese women, Asian-Pacific women with higher education pay more attention to weight control [14, 41], while Korean males were least worried about being overweight [44]. Current study found lower achievement of ideal biological and overall CVH in older individuals, which re-emphasizes the vulnerability of this subgroup. Age did not modify the SES disparities in any CVH metric among men, whereas SES disparities were lower in ideal biological factors for older women. Further research is warranted to confirm the age-modified SES disparities in biological risk profile among women.

### SES and transition from ideal CVH to suboptimal health and CVD

The concept of ideal CVH promotes primordial prevention of controlling risk factors within favorable levels and allows the feasibility of reversing individuals’ suboptimal cardiovascular health back to ideal state, avoiding the irreversible clinical CVD events and enormous medical burden. Low SES predisposes individuals to detrimental status of determinants of health that aggravate one’s CVH state. These detrimental factors include less access to timely and high-quality healthcare care [45], safe housing, hygiene, healthy food, and recreational facilities [46]. Additionally, lower SES is associated with health-compromising lifestyle (e.g., cigarette smoking) [19], a lack of coping strategies [47] against depressive, and stressful emotions. Governmental policies that aim to reduce upstream structural socioeconomic hierarchy should be considered as psychological and lifestyle factors could not fully explain SES disparities in ideal CVH [35]. Such policies may include guaranteed universal access to high-quality childcare, education, and regional affluence of facilities and resources.

## Limitations

Several limitations should be borne in mind when interpreting our findings. First, other informative SES indicators, such as regional deprivation represented by the poverty rate in neighborhoods were unavailable for constructing the latent SES that fully represent the actual situation among general Korean adults [23]. We further investigated ideal CVH associated with education and household income in isolation, and the results did not change substantially (S3 and S4 Figs in S1 File). This finding suggests SES latent was comparable in summarizing major SES characteristics. Second, misclassification in lower-medium SES might exist; the posterior probability of this class is lower than 0.70 (S1 File) indicating some uncertainty in the assignment of individuals in this category [48], which may impact the precise estimation of later analyses. Nonetheless, model fit indices and interpretability supported the four-class model with adequate membership distinction. Third, smoking, PA, and dietary intake was self-reported information that were prone to information bias and thus may impact the evaluation of genuine CVH. Fifth, our results may be due to reverse causality that participants with poor CVH were less likely to be employed in high-paying occupations and they would need more support from health security. Sixth, KNHANES participants represent a sample of noninstitutionalized population in South Korea, which limits the generalizability of our findings to other populations. Last, our finding may receive impact of time trend. We then repeated analysis separated by two periods (2007 to 2012 and 2013 to 2017) and the results showed no substantial difference between the major findings (S5, S6 Figs and S4-S7 Tables in S1 File). Despite these pitfalls, our study contributes to the literature by conducting an in-depth investigation into sex-modified SES disparities in ideal CVH using a multidimensional summary measure of SES.

## Conclusions

Taken together, low SES was strongly associated with poor achievement of ideal CVH with different patterns by sex. Optimizing CVH in Korean adults has been long overdue. Current study argues for policies that address unequal upstream distribution of power and resources to prevent shifting from ideal to suboptimal cardiovascular health and to close socioeconomic disparities in CVH and further studies are warranted to elucidate the sex-specific causal link between SES and CVH. Prevention programs that empower low-SES populations and use targeted strategies such as preventing obesity in high-SES men, promoting a healthy lifestyle for young adults, and managing biological risk factors for older adults, render opportunities to optimize the CVH and resilience of the populations against CVD.

## Supporting information

S1 File (DOCX)
