# Supplementary material for: Sex-specific associations between socioeconomic status and ideal cardiovascular health among Korean adults: The Korea National Health and Nutrition Examination Survey, 2007–2017
Source: PLoS One. 2024 Aug 15;19(8):e0307040. doi: 10.1371/journal.pone.0307040 (PMC11326625; doi:10.1371/journal.pone.0307040)
Supplement: S1 File — (DOCX) [file pone.0307040.s001.docx]

**Supplementary methods**

**Revisions of BMI and diet metrics**

BMI metric was categorized according to the Asian-Pacific recommendation and 2018 Korean Society for the Study of Obesity Guideline. Poor BMI was set as obesity (≥ 25 kg/m^2^), intermediate BMI level was set as underweight (<18.5 kg/m^2^) or overweight (23-24.9 kg/m^2^) and Ideal BMI level was within normal range (18.5-22.9 kg/m^2^). Twenty-four-hour recall survey was conducted to quantify the intake of specific food items. Five components were used for evaluating diet quality metric: consuming of fruits and vegetables (jams, juices, and starchy vegetables excluded) ≥450g/day, fish ≥198g/week, whole or mixed grains ≥ 27g/day, sodium <1,500 mg/day, and sugar-sweetened beverages ≤1 liter/week, respectively, according to 2015 Korean Dietary Reference Intake Recommendation. The original suggestion from AHA on fiber rich mixed grain intake is three 27g-equivalent servings/day. Due to the generally low intake of mixed grains among Korean population, we revised its adequate intake as 27g (1 serving)/day.

**Latent class analysis (LCA)**

Latent class analysis (LCA) is a commonly used statistical approach to distinguish population subgroups (latent classes) based on heterogeneity in a series of observable categorical features, and thus reduce data complexity. LCA uses the expectation-maximization (EM) algorithm to estimate the model parameters: 1. LCA sets random or manually-set values as initial estimates of model parameters, which include individual probabilities of belonging to each latent class (class membership probabilities) and conditional probabilities of the component variables in each latent class. 2. Expectation (E) step: LCA calculates the expected posterior probabilities of class membership for each subject in the dataset, based on the parameters set in step 1. 3. Maximization (M) step: LCA then update model parameters estimated from E step to maximize the likelihood of the complete data using expected posterior probabilities across all individuals in the dataset and updated conditional probabilities of the component variables for each generated latent class. 4. Convergence check: assessing whether the model parameters have converged or not by comparing the change in the likelihood (information criterion) between successive iterations within a defaulted-set value of 5,000. If the change is below a pre-specified threshold, the algorithm is considered to have converged, and the process stops. Otherwise, LCA return to step 2 and continue iterating. In other word, the EM algorithm is an iterative process that alternates between the E and M steps until reaching convergence.

**LCA models for current study**

We run LCAs for fitting and comparing a set of models, starting with a one-class (baseline) model and increasing classes in a stepwise fashion until model fit no longer significantly improved. This allow us to inspect the maximum posterior probability of each participant assigned to the designated classes. Final model selection should consider optimal model fit indices (including the Akaike’s Information Criterion [AIC], consist AIC [cAIC], Bayesian Information Criterion [BIC], adjusted BIC [aBIC], log likelihood [LL], G-squared likelihood ratio [G_SQUARED], and entropy), elbow plot method, maximum posterior probability, as well as the interpretability (i.e., the meaningfulness and distinctiveness) of the generated latent classes. Lower AIC, BIC and aBIC indices and higher entropy (preferably ≥0.8) indicate the appropriate class number for mutually exhaustive distinctiveness. We also presented elbow plots of statistics indices for visualization, where the elbow of the curve, the point at which the values stop decreasing, signifies favorable number of clusters. The mean posterior probabilities of latent classes reflect the certainty of posterior classification; a value of ≥0.77 indicated an acceptable certainty that participants were assigned into adequate classes. Although the 7-calss model has the least indices values (e.g., aBIC=997.8), it has a low entropy value of 0.57 and posterior probabilities of ≤0.77 in certain classes. In contrast, the elbow plot shows a turning point at the 4-class model and a less decrease in aBIC subsequently, an entropy of 0.78 that approximates 0.8 as well as generally ≥0.77 posterior probabilities in all classes (except 0.61 for the lower-medium SES class). Hence, we regard the 4-class model as an optimal fit. The four generated SES gradients also implies meaningful interpretability.

**
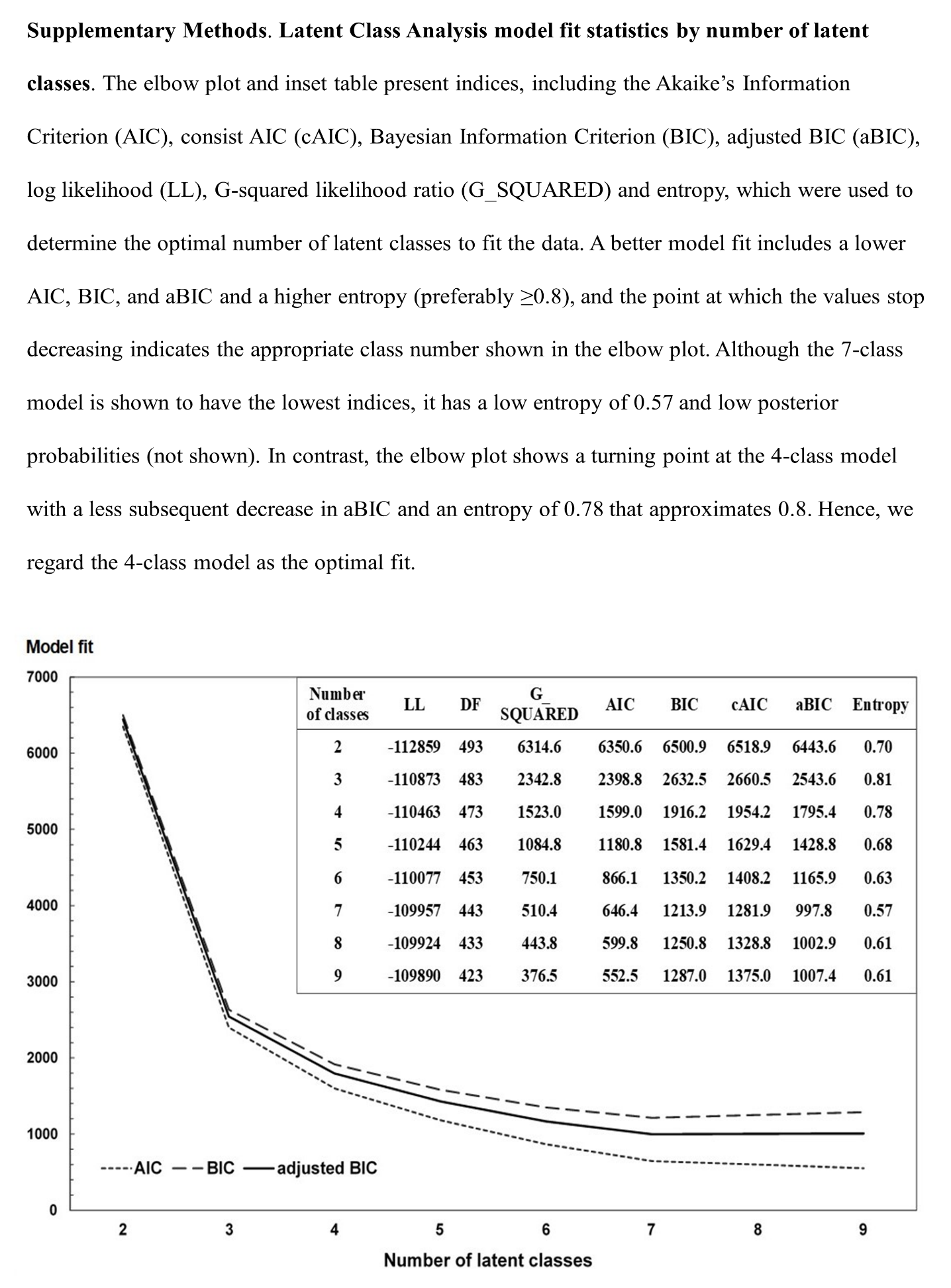
**

| **Supplementary methods. Mean posterior probabilities, prevalence of latent classes, and item-response probabilities* in three- to five-latent class models** | | | | | |
| --- | --- | --- | --- | --- | --- |
| Item | LC 1 | LC 2 | LC 3 | LC 4 | LC 5 |
|  | Three-latent-class model | | | | |
| Mean PP^†^ | **0.908** | **0.911** | **0.940** | NA | NA |
| Prevalence | 0.029 | 0.462 | 0.509 | NA | NA |
| Education 1 (≥ college) | 0.184 | 0.078 | **0.930** | NA | NA |
| Education 2 (< college) | **0.816** | **0.922** | 0.070 | NA | NA |
| Household income 1 (≥2 million won) | 0.044 | 0.023 | 0.350 | NA | NA |
| Household income 2 (<2 million won) | **0.956** | **0.977** | **0.650** | NA | NA |
| Occupation 1 (Non-manual worker) | 0.012 | 0.449 | **0.653** | NA | NA |
| Occupation 2 (Manual worker) | **0.908** | **0.551** | 0.347 | NA | NA |
| Residence area 1 ( metropolitan cities) | **0.794** | **0.740** | **0.905** | NA | NA |
| Residence area 2 ( suburb area) | 0.206 | 0.260 | 0.095 | NA | NA |
| House ownership 1 (owning ≥1 house) | 0.173 | **0.747** | **0.706** | NA | NA |
| House ownership 1 (owning no house) | **0.827** | 0.253 | 0.294 | NA | NA |
| Governmental subsidies 1 (recipient) | **0.870** | 0.025 | 0.010 | NA | NA |
| Governmental subsidies 2 (non-recipient) | 0.130 | **0.975** | **0.990** | NA | NA |
| Medicaid 1 (recipient) | **0.563** | 0.001 | 0.0002 | NA | NA |
| Medicaid 2 (non-recipient) | 0.437 | **0.999** | **1.000** | NA | NA |
| Paternal education 1 (≥ high school) | 0.198 | 0.149 | **0.563** | NA | NA |
| Paternal education 2 (< high school) | **0.802** | **0.851** | 0.437 | NA | NA |
|  | Four-latent-class model | | | | |
| Mean PP | **0.861** | **0.607** | **0.916** | **0.900** | NA |
| Prevalence | 0.024 | 0.101 | 0.437 | 0.438 | NA |
| Education 1 (≥ college) | 0.165 | 0.289 | 0.077 | **0.931** | NA |
| Education 2 (< college) | **0.835** | **0.711** | **0.923** | 0.069 | NA |
| Household income 1 (≥ 2 million won) | 0.012 | 0.111 | 0.491 | **0.681** | NA |
| Household income 2 (< 2 million won) | **0.988** | **0.889** | **0.509** | 0.319 | NA |
| Occupation 1 (Non-manual worker) | 0.041 | 0.050 | 0.022 | 0.362 | NA |
| Occupation 2 (Manual worker) | **0.959** | **0.950** | **0.978** | **0.638** | NA |
| Residence area 1 ( metropolitan cities) | **0.777** | **0.893** | **0.720** | **0.906** | NA |
| Residence area 2 ( suburb area) | 0.223 | 0.107 | 0.280 | 0.094 | NA |
| House ownership 1 (owning ≥1 house) | 0.176 | 0.007 | **0.848** | **0.738** | NA |
| House ownership 1 (owning no house) | **0.824** | **0.993** | 0.152 | 0.262 | NA |
| Governmental subsidies 1 (recipient) | **0.902** | 0.059 | 0.027 | 0.008 | NA |
| Governmental subsidies 2 (non-recipient) | 0.098 | **0.941** | **0.973** | **0.992** | NA |
| Medicaid 1 (recipient) | **0.682** | 0.001 | 0.002 | 0.0003 | NA |
| Medicaid 2 (non-recipient) | 0.318 | **0.999** | **0.998** | **1.000** | NA |
| Paternal education 1 (≥ high school) | 0.166 | 0.328 | 0.131 | **0.569** | NA |
| Paternal education 2 (< high school) | **0.834** | **0.672** | **0.869** | 0.431 | NA |
|  | Five-latent-class model | | | | |
| Mean PP | **0.833** | **0.605** | **0.660** | **0.704** | **0.927** |
| Prevalence | 0.023 | 0.109 | 0.132 | 0.298 | 0.438 |
| Education 1 (≥ college) | 0.155 | 0.310 | 0.110 | 0.001 | **0.932** |
| Education 2 (< college) | **0.845** | **0.690** | **0.890** | **0.999** | 0.068 |
| Occupation 1 (Non-manual worker) | 0.041 | 0.051 | 0.026 | 0.017 | 0.354 |
| Occupation 2 (Manual worker) | **0.959** | **0.949** | **0.974** | **0.883** | **0.646** |
| Household income 1 (≥2 million won) | 0.014 | 0.023 | 0.368 | **0.596** | **0.689** |
| Household income 2 (<2 million won) | **0.986** | **0.977** | **0.632** | 0.404 | 0.311 |
| Residence area 1 ( metropolitan cities) | **0.793** | **0.870** | **0.502** | **0.850** | **0.907** |
| Residence area 2 ( suburb area) | 0.207 | 0.130 | 0.498 | 0.150 | 0.093 |
| House ownership 1 (owning ≥1 house) | 0.153 | 0.172 | **0.895** | **0.797** | **0.738** |
| House ownership 1 (owning no house) | **0.847** | **0.828** | 0.105 | 0.203 | 0.262 |
| Governmental subsidies 1 (recipient) | **0.942** | 0.067 | 0.043 | 0.017 | 0.009 |
| Governmental subsidies 2 (non-recipient) | 0.058 | **0.933** | **0.957** | **0.983** | **0.991** |
| Medicaid 1 (recipient) | **0.726** | 0.008 | 0.005 | 0.0001 | 0.0002 |
| Medicaid 2 (non-recipient) | 0.274 | **0.992** | **0.995** | **1.000** | **1.000** |
| Paternal education 1 (≥ high school) | 0.162 | 0.320 | 0.046 | 0.179 | **0.558** |
| Paternal education 2 (< high school) | **0.838** | **0.680** | **0.954** | **0.821** | 0.442 |
| ^*^ The maximal item-response probabilities for each latent class were marked in bold. | | | | | |
| ^†^ Prevalence indicated the prevalence of each latent class. | | | | | |
| **Abbreviations**. LA, latent class; Mean PP, mean posterior probability; NA, not available. | | | | | |

| **S1 Table. Definition and revision of 7 metrics and CVH status** | | | | |
| --- | --- | --- | --- | --- |
| **CVH metrics** | **Poor** | **Intermediate** | **Ideal** | **Original or revision source** |
| **Behavioral metrics** |  |  |  |  |
| **Smoking** | Current smoker | Former smoker | Never smoker | AHA |
| **BMI (kg/m^2^)** | ≥25 | 23-25 or <18.5 | 18.5-23 | 2018 Korean Society for   the Study of Obesity   Guideline for the   Management of Obesity |
| **Physical activity, PA   (min/week)** | None | 1-149 MPA or MVPA, or 1-74 VPA | ≥ 150 MPA or MVPA, or ≥75 MVPA | AHA |
|  |  |  |  |  |
| **Diet quality** | 1. Fruits and vegetables ≥450 g/day; 2. Fish ≥198 g/week;   3.Whole grain or mixed grains ≥ 27 g (0.3 serving) /day*;   4. Sodium <1,500 mg/day; 5. Sugar-sweetened beverages ≤1 liter/week | | | AHA (*2015 Korean Dietary Reference Intake) |
|  | 0-1 component | 2-3 components | 4-5 components |  |
| **Biological metrics** |  |  |  |  |
| **Total cholesterol,**  **TC (mg/dL)** | ≥240 | 200-239 or treated  to goal (<240) | Untreated <200 | AHA |
|  |  |  |  |  |
| **Blood pressure, BP   (mmHg)** | ≥140/90 | 120-139/80-90 or treated to goal (<140/90) | Untreated <120/80 | AHA |
|  |  |  |  |  |
| **Fasting glucose,**  **FPG (mg/dL)** | ≥126 | 100-125 or treated  to goal (<126) | Untreated <100 | AHA |
| **CVH aspects** |  |  |  |  |
| **Behavioral CVH** | 0-1 behavioral metrics at ideal status | 2 behavioral metrics at ideal status | 3-4 behavioral metrics at ideal status | AHA |
| **Biological CVH** | 0-1 biological metrics at ideal status | 2 biological metrics at ideal status | 3 biological metrics at ideal status | AHA |
| **Overall CVH** | 0-2 metrics at ideal status (regardless of behavioral or biological) | 3-4 metrics  at ideal status | 5-7 metrics at ideal status | AHA |

| **S2 Table. Odds ratios (95%CI) of ideal CVH**^*^ **across SES gradients by sex** | | | | | | | | | | |
| --- | --- | --- | --- | --- | --- | --- | --- | --- | --- | --- |
| SES class | Individual metric | | | | | | | Behavioral  CVH | Biological  CVH | Overall  CVH |
|  | Smoking | PA | Diet | BMI | BP | FPG | TC |  |  |  |
|  | Male (n=12,590) | | | | | | | | | |
| Model 1† |  |  |  |  |  |  |  |  |  |  |
| High | 1.00 | 1.00 | 1.00 | 1.00 | 1.00 | 1.00 | 1.00 | 1.00 | 1.00 | 1.00 |
| Higher-  medium | 0.65  (0.58-0.73) | 0.96  (0.87-1.06) | 0.90  (0.82-1.00) | 1.14  (1.03-1.26) | 0.97  (0.87-1.07) | 0.86  (0.78-0.95) | 1.22  (1.10-1.34) | 0.84  (0.72-0.98) | 0.97  (0.85-1.10) | 0.89  (0.77-1.02) |
| Lower-  medium | 0.48  (0.39-0.59) | 0.86  (0.74-1.00) | 0.82  (0.70-0.95) | 1.24  (1.07-1.43) | 0.97  (0.84-1.12) | 0.91  (0.78-1.06) | 1.16  (1.00-1.34) | 0.65  (0.50-0.85) | 1.03  (0.87-1.23) | 0.83  (0.67-1.03) |
| Low | 0.66  (0.45-0.98) | 0.47  (0.34-0.64) | 0.62  (0.46-0.85) | 1.51  (1.14-2.00) | 0.77  (0.57-1.02) | 0.58  (0.43-0.79) | 0.96  (0.73-1.27) | 0.52  (0.29-0.94) | 0.84  (0.57-1.24) | 0.76  (0.47-1.24) |
| P-trend | <.0001 | 0.0002 | 0.0002 | <.0001 | 0.185 | 0.001 | 0.014 | 0.001 | 0.772 | 0.128 |
| Model 2^‡^ |  |  |  |  |  |  |  |  |  |  |
| High | 1.00 | 1.00 | 1.00 | 1.00 | 1.00 | 1.00 | 1.00 | 1.00 | 1.00 | 1.00 |
| Higher-  medium | 0.67  (0.59-0.75) | 0.96  (0.87-1.06) | 0.92  (0.83-1.01) | 1.15  (1.04-1.27) | 1.01  (0.91-1.11) | 0.88  (0.79-0.98) | 1.22  (1.11-1.34) | 0.85  (0.72-1.01) | 0.97  (0.85-1.10) | 0.92  (0.80-1.07) |
| Lower-  medium | 0.49  (0.40-0.60) | 0.86  (0.74-1.00) | 0.84  (0.72-0.98) | 1.23  (1.06-1.42) | 0.97  (0.84-1.13) | 0.89  (0.76-1.05) | 1.16  (1.00-1.34) | 0.70  (0.53-0.92) | 1.03  (0.87-1.23) | 0.87  (0.69-1.09) |
| Low | 0.65  (0.44-0.97) | 0.47  (0.34-0.64) | 0.73  (0.53-1.00) | 1.45  (1.07-1.95) | 0.86  (0.64-1.16) | 0.60  (0.43-0.82) | 0.97  (0.73-1.27) | 0.58  (0.32-1.04) | 0.84  (0.57-1.24) | 0.83  (0.51-1.34) |
| P-trend | <.0001 | 0.001 | 0.004 | 0.0002 | 0.506 | 0.001 | 0.017 | 0.001 | 0.995 | 0.233 |
|  | Female (n=18,551) | | | | | | | | | |
| Model 1 |  |  |  |  |  |  |  |  |  |  |
| High | 1.00 | 1.00 | 1.00 | 1.00 | 1.00 | 1.00 | 1.00 | 1.00 | 1.00 | 1.00 |
| Higher-  medium | 0.71  (0.61-0.83) | 1.05  (0.96-1.16) | 0.85  (0.78-0.93) | 0.66  (0.61-0.72) | 0.67  (0.61-0.74) | 0.67  (0.59-0.74) | 1.05  (0.96-1.14) | 0.73  (0.66-0.80) | 0.78  (0.72-0.86) | 0.69  (0.63-0.76) |
| Lower-  medium | 0.40  (0.34-0.47) | 0.76  (0.67-0.87) | 0.70  (0.62-0.80) | 0.68  (0.60-0.76) | 0.65  (0.57-0.74) | 0.62  (0.53-0.72) | 0.90  (0.79-1.02) | 0.51  (0.44-0.58) | 0.77  (0.68-0.87) | 0.53  (0.46-0.60) |
| Low | 0.24  (0.18-0.32) | 0.58  (0.45-0.75) | 0.53  (0.41-0.69) | 0.62  (0.49-0.78) | 0.72  (0.57-0.92) | 0.42  (0.33-0.54) | 0.97  (0.78-1.22) | 0.36  (0.26-0.49) | 0.78  (0.61-1.01) | 0.43  (0.33-0.55) |
| P-trend | <.0001 | <.0001 | <.0001 | <.0001 | <.0001 | <.0001 | 0.275 | <.0001 | <.0001 | <.0001 |
| Model 2 |  |  |  |  |  |  |  |  |  |  |
| High | 1.00 | 1.00 | 1.00 | 1.00 | 1.00 | 1.00 | 1.00 | 1.00 | 1.00 | 1.00 |
| Higher-  medium | 0.79  (0.67-0.93) | 1.05  (0.96-1.16) | 0.86  (0.78-0.94) | 0.68  (0.62-0.74) | 0.69  (0.63-0.77) | 0.68  (0.61-0.77) | 1.06  (0.97-1.16) | 0.76  (0.69-0.83) | 0.81  (0.74-0.88) | 0.72  (0.66-0.79) |
| Lower-  medium | 0.47  (0.39-0.56) | 0.76  (0.67-0.87) | 0.72  (0.64-0.83) | 0.70  (0.62-0.79) | 0.70  (0.60-0.80) | 0.63  (0.54-0.73) | 0.92  (0.81-1.04) | 0.55  (0.47-0.63) | 0.80  (0.70-0.91) | 0.57  (0.50-0.65) |
| Low | 0.43  (0.32-0.59) | 0.58  (0.45-0.75) | 0.60  (0.46-0.78) | 0.70  (0.55-0.88) | 0.85  (0.66-1.09) | 0.44  (0.34-0.57) | 1.09  (0.86-1.36) | 0.43  (0.32-0.60) | 0.96  (0.74-1.24) | 0.55  (0.43-0.71) |
| P-trend | <.0001 | <.0001 | <.0001 | <.0001 | <.0001 | <.0001 | 0.775 | <.0001 | 0.0003 | <.0001 |
| ^*^ Ideal behavioral CVH was defined as having 3-4 out of 4 behavioral metrics (smoking, PA, diet, and BMI) at ideal status. Ideal biological CVH was defined as having 3 biological metrics (blood pressure, fasting plasma glucose, and total cholesterol) at ideal status. Ideal overall CVH was defined as having 5-7 out of 7  total metrics at ideal status  † Model 1 was age-adjusted | | | | | | | | | | |
| ^‡^ Model 2 further adjusted for partnership, CCI, depressive symptoms, stress, high risk of drinking, and health checkup | | | | | | | | | | |
| **Abbreviations**. CVH, cardiovascular health; SES, socio-economic status; PA, physical activities; BMI, body mass index; BP, blood pressure; FPG, fasting | | | | | | | | | | |
| plasma glucose; TC, total cholesterol | | | | | | | | | | |

| \| **S3 Table. Multivariate-adjusted Odds ratios (95%CI)* of ideal CVH**^†^ **across SES gradients by age category** \| \| \| \| \| \| \| \| --- \| --- \| --- \| --- \| --- \| --- \| --- \| \| Population \| Ideal CVH \| SES class \| \| \| \| P-trend \| \| High \| Higher-medium \| Lower-medium \| Low \| \| Total \| Smoking \| 1.00 \| 0.75 (0.69-0.82) \| 0.46 (0.40-0.53) \| 0.47 (0.36-0.62) \| <.0001 \| \| (n=31,141) \| PA \| 1.00 \| 1.02 (0.95-1.10) \| 0.82 (0.74-0.91) \| 0.54 (0.44-0.67) \| <.0001 \| \|  \| Diet \| 1.00 \| 0.89 (0.83-0.95) \| 0.78 (0.70-0.87) \| 0.66 (0.54-0.81) \| <.0001 \| \|  \| BMI \| 1.00 \| 0.85 (0.80-0.91) \| 0.91 (0.83-1.00) \| 0.98 (0.80-1.18) \| 0.011 \| \|  \| BP \| 1.00 \| 0.82 (0.77-0.88) \| 0.85 (0.77-0.95) \| 0.94 (0.78-1.13) \| 0.0001 \| \|  \| FPG \| 1.00 \| 0.80 (0.74-0.86) \| 0.78 (0.69-0.87) \| 0.53 (0.43-0.66) \| <.0001 \| \|  \| TC \| 1.00 \| 1.10 (1.03-1.18) \| 1.04 (0.94-1.14) \| 1.06 (0.89-1.27) \| 0.119 \| \|  \| Behavioral CVH \| 1.00 \| 0.80 (0.74-0.87) \| 0.59 (0.52-0.67) \| 0.46 (0.35-0.62) \| <.0001 \| \|  \| Biological CVH \| 1.00 \| 0.86 (0.80-0.93) \| 0.89 (0.80-0.98) \| 0.98 (0.79-1.21) \| 0.006 \| \|  \| Overall CVH \| 1.00 \| 0.80 (0.74-0.86) \| 0.66 (0.58-0.74) \| 0.64 (0.50-0.81) \| <.0001 \| \| Male \| Smoking \| 1.00 \| 0.58 (0.50-0.69) \| 0.45 (0.35-0.57) \| 0.88 (0.53-1.45) \| <.0001 \| \| age 25-49 \| PA \| 1.00 \| 0.98 (0.85-1.12) \| 0.93 (0.78-1.10) \| 0.59 (0.39-0.91) \| 0.096 \| \| (n=7,651) \| Diet \| 1.00 \| 0.90 (0.78-1.03) \| 0.85 (0.71-1.04) \| 0.70 (0.44-1.11) \| 0.024 \| \|  \| BMI \| 1.00 \| 1.08 (0.95-1.24) \| 1.29 (1.08-1.54) \| 1.67 (1.08-2.56) \| 0.001 \| \|  \| BP \| 1.00 \| 1.02 (0.89-1.16) \| 1.03 (0.87-1.22) \| 1.12 (0.74-1.67) \| 0.608 \| \|  \| FPG \| 1.00 \| 0.83 (0.72-0.96) \| 0.99 (0.81-1.20) \| 0.76 (0.44-1.30) \| 0.154 \| \|  \| TC \| 1.00 \| 1.15 (1.01-1.31) \| 1.11 (0.93-1.32) \| 1.35 (0.87-2.10) \| 0.034 \| \|  \| Behavioral CVH \| 1.00 \| 0.82 (0.65-1.04) \| 0.66 (0.49-0.91) \| 0.67 (0.28-1.61) \| 0.006 \| \|  \| Biological CVH \| 1.00 \| 0.95 (0.81-1.10) \| 1.02 (0.84-1.24) \| 1.33 (0.79-2.25) \| 0.759 \| \|  \| Overall CVH \| 1.00 \| 0.87 (0.72-1.05) \| 0.89 (0.70-1.14) \| 1.44 (0.83-2.52) \| 0.502 \| \| Male \| Smoking \| 1.00 \| 0.82 (0.68-0.98) \| 0.82 (0.56-1.22) \| 0.72 (0.39-1.33) \| 0.061 \| \| age 50-64 \| PA \| 1.00 \| 0.90 (0.77-1.04) \| 0.77 (0.58-1.03) \| 0.43 (0.27-0.69) \| 0.001 \| \| (n=4,939) \| Diet \| 1.00 \| 0.94 (0.81-1.09) \| 0.80 (0.61-1.05) \| 0.74 (0.48-1.16) \| 0.069 \| \|  \| BMI \| 1.00 \| 1.24 (1.05-1.46) \| 1.09 (0.80-1.47) \| 1.34 (0.88-2.06) \| 0.065 \| \|  \| BP \| 1.00 \| 0.95 (0.81-1.11) \| 0.76 (0.57-1.02) \| 0.57 (0.35-0.91) \| 0.015 \| \|  \| FPG \| 1.00 \| 0.90 (0.78-1.05) \| 0.87 (0.66-1.17) \| 0.72 (0.46-1.13) \| 0.088 \| \|  \| TC \| 1.00 \| 1.32 (1.14-1.53) \| 1.34 (1.01-1.79) \| 0.78 (0.52-1.17) \| 0.122 \| \|  \| Behavioral CVH \| 1.00 \| 0.94 (0.74-1.20) \| 0.88 (0.54-1.43) \| 0.56 (0.27-1.18) \| 0.224 \| \|  \| Biological CVH \| 1.00 \| 1.11 (0.87-1.41) \| 1.14 (0.76-1.71) \| 0.59 (0.30-1.16) \| 0.958 \| \|  \| Overall CVH \| 1.00 \| 1.17 (0.91-1.50) \| 0.88 (0.53-1.47) \| 0.32 (0.11-0.94) \| 0.371 \| \| Female \| Smoking \| 1.00 \| 0.70 (0.58-0.83) \| 0.47 (0.39-0.56) \| 0.47 (0.32-0.69) \| <.0001 \| \| age 25-49 \| PA \| 1.00 \| 1.07 (0.96-1.20) \| 0.79 (0.68-0.92) \| 0.56 (0.40-0.78) \| 0.001 \| \| (n= 11,797) \| Diet \| 1.00 \| 0.88 (0.79-0.98) \| 0.67 (0.57-0.78) \| 0.59 (0.42-0.85) \| <.0001 \| \|  \| BMI \| 1.00 \| 0.71 (0.64-0.78) \| 0.72 (0.63-0.83) \| 0.71 (0.64-0.78) \| <.0001 \| \|  \| BP \| 1.00 \| 0.69 (0.61-0.78) \| 0.69 (0.58-0.82) \| 1.01 (0.71-1.44) \| <.0001 \| \|  \| FPG \| 1.00 \| 0.63 (0.55-0.73) \| 0.62 (0.51-0.75) \| 0.44 (0.31-0.61) \| <.0001 \| \|  \| TC \| 1.00 \| 1.01 (0.91-1.13) \| 0.89 (0.77-1.03) \| 1.19 (0.87-1.62) \| 0.637 \| \|  \| Behavioral CVH \| 1.00 \| 0.80 (0.72-0.89) \| 0.52 (0.44-0.61) \| 0.49 (0.33-0.72) \| <.0001 \| \|  \| Biological CVH \| 1.00 \| 1.01 (0.75-1.36) \| 0.80 (0.69-0.91) \| 0.82 (0.74-0.90) \| 0.001 \| \|  \| Overall CVH \| 1.00 \| 0.73 (0.66-0.81) \| 0.57 (0.49-0.66) \| 0.61 (0.45-0.82) \| <.0001 \| \| Female \| Smoking \| 1.00 \| 1.54 (1.04-2.30) \| 0.72 (0.44-1.18) \| 0.53 (0.31-0.93) \| 0.009 \| \| age 50-64 \| PA \| 1.00 \| 0.95 (0.79-1.13) \| 0.60 (0.45-0.80) \| 0.47 (0.30-0.74) \| <.0001 \| \| (n=6,754) \| Diet \| 1.00 \| 0.82 (0.69-0.96) \| 0.83 (0.65-1.06) \| 0.52 (0.35-0.78) \| 0.002 \| \|  \| BMI \| 1.00 \| 0.57 (0.49-0.68) \| 0.57 (0.44-0.73) \| 0.57 (0.39-0.82) \| <.0001 \| \|  \| BP \| 1.00 \| 0.71 (0.60-0.83) \| 0.69 (0.54-0.89) \| 0.73 (0.51-1.05) \| 0.002 \| \|  \| FPG \| 1.00 \| 0.81 (0.68-0.97) \| 0.75 (0.56-0.99) \| 0.67 (0.46-0.98) \| 0.010 \| \|  \| TC \| 1.00 \| 1.23 (1.05-1.44) \| 1.02 (0.78-1.33) \| 1.01 (0.71-1.45) \| 0.742 \| \|  \| Behavioral CVH \| 1.00 \| 0.65 (0.55-0.77) \| 0.55 (0.42-0.73) \| 0.26 (0.15-0.44) \| <.0001 \| \|  \| Biological CVH \| 1.00 \| 0.75 (0.60-0.93) \| 0.74 (0.52-1.05) \| 0.71 (0.42-1.20) \| 0.037 \| \|  \| Overall CVH \| 1.00 \| 0.68 (0.57-0.82) \| 0.51 (0.38-0.70) \| 0.31 (0.17-0.57) \| <.0001 \| \| * Full model adjusted for age, partnership, CCI, depressive symptoms, stress, high risk of drinking, and health checkup; sex was additionally adjusted for total population \| \| \| \| \| \| \| \| † Ideal behavioral CVH was defined as having 3-4 out of 4 behavioral metrics (smoking, PA, diet, and BMI) at ideal status. Ideal biological CVH was defined as having 3 biological metrics (blood pressure, fasting plasma glucose, and total cholesterol) at ideal status. Ideal overall CVH was defined as having 5-7 out of 7 total metrics at ideal status \| \| \| \| \| \| \| \| **Abbreviations.** CVH, cardiovascular health; SES, socio-economic status; PA, physical activities; BMI, body mass \| \| \| \| \| \| \| \| index; BP, blood pressure; FPG, fasting plasma glucose; TC, total cholesterol \| \| \| \| \| \| \| |
| --- | --- | --- | --- | --- | --- | --- | --- | --- | --- | --- | --- | --- | --- | --- | --- | --- | --- | --- | --- | --- | --- | --- | --- | --- | --- | --- | --- | --- | --- | --- | --- | --- | --- | --- | --- | --- | --- | --- | --- | --- | --- | --- | --- | --- | --- | --- | --- | --- | --- | --- | --- | --- | --- | --- | --- | --- | --- | --- | --- | --- | --- | --- | --- | --- | --- | --- | --- | --- | --- | --- | --- | --- | --- | --- | --- | --- | --- | --- | --- | --- | --- | --- | --- | --- | --- | --- | --- | --- | --- | --- | --- | --- | --- | --- | --- | --- | --- | --- | --- | --- | --- | --- | --- | --- | --- | --- | --- | --- | --- | --- | --- | --- | --- | --- | --- | --- | --- | --- | --- | --- | --- | --- | --- | --- | --- | --- | --- | --- | --- | --- | --- | --- | --- | --- | --- | --- | --- | --- | --- | --- | --- | --- | --- | --- | --- | --- | --- | --- | --- | --- | --- | --- | --- | --- | --- | --- | --- | --- | --- | --- | --- | --- | --- | --- | --- | --- | --- | --- | --- | --- | --- | --- | --- | --- | --- | --- | --- | --- | --- | --- | --- | --- | --- | --- | --- | --- | --- | --- | --- | --- | --- | --- | --- | --- | --- | --- | --- | --- | --- | --- | --- | --- | --- | --- | --- | --- | --- | --- | --- | --- | --- | --- | --- | --- | --- | --- | --- | --- | --- | --- | --- | --- | --- | --- | --- | --- | --- | --- | --- | --- | --- | --- | --- | --- | --- | --- | --- | --- | --- | --- | --- | --- | --- | --- | --- | --- | --- | --- | --- | --- | --- | --- | --- | --- | --- | --- | --- | --- | --- | --- | --- | --- | --- | --- | --- | --- | --- | --- | --- | --- | --- | --- | --- | --- | --- | --- | --- | --- | --- | --- | --- | --- | --- | --- | --- | --- | --- | --- | --- | --- | --- | --- | --- | --- | --- | --- | --- | --- | --- | --- | --- | --- | --- | --- | --- | --- | --- | --- | --- | --- | --- | --- | --- | --- | --- | --- | --- | --- | --- | --- | --- | --- | --- | --- | --- | --- | --- | --- | --- | --- | --- | --- | --- | --- | --- | --- | --- | --- | --- | --- | --- | --- | --- | --- | --- | --- | --- | --- | --- | --- | --- | --- | --- | --- | --- | --- | --- | --- | --- | --- | --- | --- | --- | --- | --- | --- | --- | --- | --- | --- | --- | --- | --- | --- | --- | --- | --- | --- | --- | --- | --- | --- | --- | --- | --- | --- | --- | --- | --- | --- | --- | --- | --- | --- | --- | --- |

| **S4 Table. Odds ratios (95%CI) of achieving ideal CVH status* associated with socio-demographics, SES gradients, and lifestyle factors among total population 2007-2012** | | | | | | | | | | | | | | | | | |
| --- | --- | --- | --- | --- | --- | --- | --- | --- | --- | --- | --- | --- | --- | --- | --- | --- | --- |
| Item | Ideal overall CVH | | | | |  | Ideal behavioral CVH | | | | |  | Ideal biological CVH | | | | |
|  | Model 1^†^ | |  | Model 2^‡^ | |  | Model 1 | |  | Model 2 | |  | Model 1 | |  | Model 2 | |
|  | OR | (95%CI) |  | OR | (95%CI) |  | OR | (95%CI) |  | OR | (95%CI) |  | OR | (95%CI) |  | OR | (95%CI) |
| Age per 10 year | 0.66 | (0.64-0.69) |  | 0.76 | (0.71-0.81) |  | 1.07 | (1.03-1.12) |  | 1.15 | (1.07-1.23) |  | 0.44 | (0.42-0.46) |  | 0.49 | (0.46-0.52) |
| Age group |  |  |  |  |  |  |  |  |  |  |  |  |  |  |  |  |  |
| 25-49 | 1.00 |  |  | 1.00 |  |  | 1.00 |  |  | 1.00 |  |  | 1.00 |  |  | 1.00 |  |
| 50-64 | 0.41 | (0.37-0.45) |  | 0.70 | (0.60-0.83) |  | 1.12 | (1.02-1.23) |  | 1.26 | (1.07-1.48) |  | 0.20 | (0.18-0.22) |  | 0.48 | (0.40-0.56) |
| Sex |  |  |  |  |  |  |  |  |  |  |  |  |  |  |  |  |  |
| Male | 1.00 |  |  | 1.00 |  |  | 1.00 |  |  | 1.00 |  |  | 1.00 |  |  | 1.00 |  |
| Female | 4.15 | (3.78-4.54) |  | 3.97 | (3.60-4.38) |  | 3.46 | (3.11-3.84) |  | 3.33 | (2.98-3.73) |  | 2.80 | (2.57-3.05) |  | 2.60 | (2.38-2.85) |
| SES gradient |  |  |  |  |  |  |  |  |  |  |  |  |  |  |  |  |  |
| High | 1.00 |  |  | 1.00 |  |  | 1.00 |  |  | 1.00 |  |  | 1.00 |  |  | 1.00 |  |
| Higher-medium | 0.76 | (0.69-0.84) |  | 0.79 | (0.71-0.88) |  | 0.80 | (0.72-0.90) |  | 0.83 | (0.74-0.93) |  | 0.80 | (0.72-0.88) |  | 0.82 | (0.74-0.90) |
| Lower-medium | 0.66 | (0.56-0.77) |  | 0.68 | (0.58-0.80) |  | 0.59 | (0.50-0.70) |  | 0.62 | (0.52-0.74) |  | 0.85 | (0.74-0.98) |  | 0.86 | (0.75-0.99) |
| Low | 0.58 | (0.42-0.78) |  | 0.67 | (0.49-0.91) |  | 0.49 | (0.34-0.72) |  | 0.55 | (0.37-0.81) |  | 0.72 | (0.54-0.96) |  | 0.85 | (0.64-1.14) |
| Cohabitation |  |  |  |  |  |  |  |  |  |  |  |  |  |  |  |  |  |
| Living with a partner | 1.00 |  |  | 1.00 |  |  | 1.00 |  |  | 1.00 |  |  | 1.00 |  |  | 1.00 |  |
| Single, divorced, or   widowed | 0.93 | (0.83-1.05) |  | 0.92 | (0.84-1.00) |  | 0.93 | (0.82-1.06) |  | 0.99 | (0.87-1.13) |  | 0.77 | (0.69-0.86) |  | 0.82 | (0.73-0.91) |
| CCI score | 0.76 | (0.70-0.83) |  | 0.78 | (0.73-0.83) |  | 0.90 | (0.84-0.96) |  | 0.90 | (0.84-0.96) |  | 0.76 | (0.70-0.83) |  | 0.78 | (0.72-0.85) |
| Depression |  |  |  |  |  |  |  |  |  |  |  |  |  |  |  |  |  |
| Yes vs. no | 0.84 | (0.67-1.05) |  | 0.79 | (0.66-0.94) |  | 0.79 | (0.63-0.99) |  | 0.86 | (0.68-1.09) |  | 0.80 | (0.64-1.00) |  | 0.87 | (0.70-1.09) |
| Stress |  |  |  |  |  |  |  |  |  |  |  |  |  |  |  |  |  |
| Yes vs. no | 0.86 | (0.78-0.94) |  | 0.82 | (0.76-0.88) |  | 0.86 | (0.77-0.95) |  | 0.89 | (0.80-0.99) |  | 0.87 | (0.80-0.95) |  | 0.90 | (0.82-0.98) |
| High-risk drinking |  |  |  |  |  |  |  |  |  |  |  |  |  |  |  |  |  |
| Yes vs. no | 0.50 | (0.43-0.60) |  | 0.47 | (0.42-0.54) |  | 0.55 | (0.45-0.68) |  | 0.56 | (0.46-0.69) |  | 0.58 | (0.50-0.67) |  | 0.58 | (0.51-0.67) |
| Health checkup |  |  |  |  |  |  |  |  |  |  |  |  |  |  |  |  |  |
| No vs. yes | 0.85 | (0.78-0.93) |  | 0.86 | (0.80-0.92) |  | 0.79 | (0.72-0.87) |  | 0.83 | (0.75-0.92) |  | 0.92 | (0.84-.1.00) |  | 0.95 | (0.87-1.04) |
| ^* *^ Ideal behavioral CVH was defined as having 3-4 out of 4 behavioral metrics (smoking, PA, diet, and BMI) at ideal status. Ideal biological CVH was defined as having 3 biological metrics (blood pressure, fasting plasma glucose, and total cholesterol) at ideal status. Ideal overall CVH was defined as having 5-7 out of 7  total metrics at ideal status  † Model 1 was age-adjusted | | | | | | | | | | | | | | | | | |
| ^‡^ Model 2 further adjusted for partnership, CCI, depressive symptoms, stress, high risk of drinking, and health checkup | | | | | | | | | | | | | | | | | |
| **Abbreviations**. CVH, cardiovascular health; SES, socio-economic status; CCI, Charlson Comorbidity Index | | | | | | | | | | | | | | | | | |

| **S5 Table. Odds ratios (95%CI) of achieving ideal CVH status* associated with socio-demographics, SES gradients, and lifestyle factors among total population 2013-2017** | | | | | | | | | | | | | | | | | |
| --- | --- | --- | --- | --- | --- | --- | --- | --- | --- | --- | --- | --- | --- | --- | --- | --- | --- |
| Item | Ideal overall CVH | | | | |  | Ideal behavioral CVH | | | | |  | Ideal biological CVH | | | | |
|  | Model 1^†^ | |  | Model 2^‡^ | |  | Model 1 | |  | Model 2 | |  | Model 1 | |  | Model 2 | |
|  | OR | (95%CI) |  | OR | (95%CI) |  | OR | (95%CI) |  | OR | (95%CI) |  | OR | (95%CI) |  | OR | (95%CI) |
| Age per 10 year | 0.70 | (0.65-0.76) |  | 0.76 | (0.71-0.81) |  | 1.03 | (0.98-1.08) |  | 1.09 | (1.01-1.18) |  | 0.44 | (0.42-0.47) |  | 0.50 | (0.46-0.53) |
| Age group |  |  |  |  |  |  |  |  |  |  |  |  |  |  |  |  |  |
| 25-49 | 1.00 |  |  | 1.00 |  |  | 1.00 |  |  | 1.00 |  |  | 1.00 |  |  | 1.00 |  |
| 50-64 | 0.67 | (0.57-0.79) |  | 0.70 | (0.60-0.83) |  | 1.01 | (0.91-1.13) |  | 1.02 | (0.86-1.21) |  | 0.20 | (0.18-0.22) |  | 0.42 | (0.35-0.50) |
| Sex |  |  |  |  |  |  |  |  |  |  |  |  |  |  |  |  |  |
| Male | 1.00 |  |  | 1.00 |  |  | 1.00 |  |  | 1.00 |  |  | 1.00 |  |  | 1.00 |  |
| Female | 5.33 | (4.76-5.96) |  | 5.09 | (4.53-5.73) |  | 4.21 | (3.73-4.77) |  | 4.12 | (3.60-4.68) |  | 2.94 | (2.66-3.25) |  | 2.66 | (2.40-2.96) |
| SES gradient |  |  |  |  |  |  |  |  |  |  |  |  |  |  |  |  |  |
| High | 1.00 |  |  | 1.00 |  |  | 1.00 |  |  | 1.00 |  |  | 1.00 |  |  | 1.00 |  |
| Higher-medium | 0.69 | (0.61-0.78) |  | 0.73 | (0.64-0.82) |  | 0.70 | (0.62-0.79) |  | 0.72 | (0.63-0.81) |  | 0.78 | (0.64-1.18) |  | 0.81 | (0.72-0.92) |
| Lower-medium | 0.56 | (0.46-0.67) |  | 0.60 | (0.50-0.72) |  | 0.51 | (0.41-0.62) |  | 0.54 | (0.44-0.66) |  | 0.84 | (0.72-0.99) |  | 0.86 | (0.74-1.01) |
| Low | 0.45 | (0.31-0.64) |  | 0.56 | (0.39-0.82) |  | 0.33 | (0.21-0.50) |  | 0.37 | (0.24-0.58) |  | 0.87 | (0.64-1.18) |  | 1.06 | (0.77-1.46) |
| Cohabitation |  |  |  |  |  |  |  |  |  |  |  |  |  |  |  |  |  |
| Living with a partner | 1.00 |  |  | 1.00 |  |  | 1.00 |  |  | 1.00 |  |  | 1.00 |  |  | 1.00 |  |
| Single, divorced, or   widowed | 0.83 | (0.73-0.94) |  | 0.93 | (0.82-1.06) |  | 0.95 | (0.83-1.09) |  | 1.06 | (0.92-1.22) |  | 0.81 | (0.71-0.91) |  | 0.86 | (0.75-0.97) |
| CCI score | 0.78 | (0.72-0.85) |  | 0.80 | (0.73-0.87) |  | 0.95 | (0.88-1.04) |  | 0.97 | (0.89-1.05) |  | 0.78 | (0.72-0.85) |  | 0.79 | (0.73-0.86) |
| Depression |  |  |  |  |  |  |  |  |  |  |  |  |  |  |  |  |  |
| Yes vs. no | 0.62 | (0.47-0.82) |  | 0.76 | (0.57-1.00) |  | 0.70 | (0.53-0.93) |  | 0.86 | (0.65-1.15) |  | 0.71 | (0.54-0.93) |  | 0.78 | (0.59-1.03) |
| Stress |  |  |  |  |  |  |  |  |  |  |  |  |  |  |  |  |  |
| Yes vs. no | 0.74 | (0.66-0.82) |  | 0.78 | (0.70-0.87) |  | 0.69 | (0.60-0.78) |  | 0.71 | (0.63-0.81) |  | 0.90 | (0.81-1.00) |  | 0.93 | (0.83-1.03) |
| High-risk drinking |  |  |  |  |  |  |  |  |  |  |  |  |  |  |  |  |  |
| Yes vs. no | 0.41 | (0.34-0.50) |  | 0.43 | (0.35-0.53) |  | 0.47 | (0.37-0.59) |  | 0.50 | (0.40-0.64) |  | 0.46 | (0.39-0.55) |  | 0.47 | (0.39-0.66) |
| Health checkup |  |  |  |  |  |  |  |  |  |  |  |  |  |  |  |  |  |
| No vs. yes | 0.76 | (0.68-0.84) |  | 0.83 | (0.74-0.92) |  | 0.78 | (0.70-0.87) |  | 0.84 | (0.75-0.94) |  | 0.95 | (0.85-.1.05) |  | 1.00 | (0.90-1.12) |
| ^* *^ Ideal behavioral CVH was defined as having 3-4 out of 4 behavioral metrics (smoking, PA, diet, and BMI) at ideal status. Ideal biological CVH was defined as having 3 biological metrics (blood pressure, fasting plasma glucose, and total cholesterol) at ideal status. Ideal overall CVH was defined as having 5-7 out of 7  total metrics at ideal status | | | | | | | | | | | | | | | | | |
| ^†^ Model 1 was age-adjusted  ^‡^ Model 2 further adjusted for partnership, CCI, depressive symptoms, stress, high risk of drinking, and health checkup | | | | | | | | | | | | | | | | | |
| **Abbreviations**. CVH, cardiovascular health; SES, socio-economic status; CCI, Charlson Comorbidity Index | | | | | | | | | | | | | | | | | |

| **S6 Table.**  **Relative inequalities (RII, 95%CI) of achieving ideal CVH^*^ by sex and age category 2007-2012** | | | | | | | | | | | | |
| --- | --- | --- | --- | --- | --- | --- | --- | --- | --- | --- | --- | --- |
|  | Total |  | By sex†^‡^ | |  | By age category^§^ | |  | By sex and age category^‡^ | | | |
|  | population^†^ |  | Male | Female |  | 25-49 years | 50-64 years |  | 25-49 years | 50-64 years male | 25-49 years | 50-64 years |
|  |  |  |  |  |  |  |  |  | male |  | female | female |
| CVH metrics |  |  |  |  |  |  |  |  |  |  |  |  |
| Smoking | 1.23 |  | 1.18 | 3.38 |  | 1.28 | 1.12 |  | 1.21 | 1.10 | 3.36 | 3.80 |
|  | (1.19-1.27) |  | (1.14-1.22) | (2.80-4.08) |  | (1.22-1.34) | (1.06-1.18) |  | (1.15-1.27) | (1.04-1.16) | (2.77-4.08) | (1.96-7.34) |
| PA | 1.12 |  | 1.14 | 1.11 |  | 1.08 | 1.22 |  | 1.07 | 1.25 | 1.09 | 1.19 |
|  | (1.08-1.17) |  | (1.06-1.22) | (1.06-1.16) |  | (1.03-1.14) | (1.14-1.30) |  | (0.98-1.18) | (1.13-1.38) | (1.03-1.15) | (1.10-1.29) |
| Diet | 1.12 |  | 1.09 | 1.15 |  | 1.11 | 1.18 |  | 1.07 | 1.17 | 1.14 | 1.18 |
|  | (1.09-1.16) |  | (1.04-1.15) | (1.11-1.19) |  | (1.07-1.15) | (1.10-1.26) |  | (1.01-1.14) | (1.06-1.30) | (1.10-1.19) | (1.08-1.29) |
| BMI | 1.04 |  | 0.90 | 1.34 |  | 1.03 | 1.04 |  | 0.9 | 0.90 | 1.37 | 1.30 |
|  | (1.00-1.08) |  | (0.86-0.95) | (1.26-1.43) |  | (0.98-1.09) | (0.98-1.11) |  | (0.84-0.96) | (0.84-0.98) | (1.27-1.49) | (1.18-1.44) |
| BP | 1.14 |  | 1.07 | 1.30 |  | 1.13 | 1.15 |  | 1.01 | 1.11 | 1.59 | 1.21 |
|  | (1.08-1.19) |  | (1.00-1.14) | (1.20-1.42) |  | (1.05-1.23) | (1.08-1.22) |  | (0.92-1.11) | (1.03-1.20) | (1.38-1.83) | (1.09-1.33) |
| FPG | 1.32 |  | 1.18 | 1.73 |  | 1.40 | 1.24 |  | 1.15 | 1.19 | 2.09 | 1.40 |
|  | (1.22-1.42) |  | (1.07-1.30) | (1.52-1.97) |  | (1.24-1.58) | (1.12-1.38) |  | (0.98-1.34) | (1.05-1.35) | (1.73-2.52) | (1.17-1.67) |
| TC | 0.94 |  | 0.85 | 1.01 |  | 0.94 | 0.91 |  | 0.85 | 0.84 | 1.09 | 0.97 |
|  | (0.89-0.99) |  | (0.78-0.93) | (0.94-1.09) |  | (0.87-1.03) | (0.85-0.99) |  | (0.75-0.95) | (0.73-0.96) | (0.97-1.23) | (0.88-1.06) |
| CVH status |  |  |  |  |  |  |  |  |  |  |  |  |
| Behavioral CVH | 1.08 |  | 1.04 | 1.27 |  | 1.08 | 1.09 |  | 1.04 | 1.04 | 1.27 | 1.31 |
|  | (1.06-1.11) |  | (1.02-1.07) | (1.22-1.32) |  | (1.06-1.11) | (1.05-1.12) |  | (1.01-1.07) | (1.00-1.08) | (1.21-1.32) | (1.22-1.40) |
| Biological CVH | 1.02 |  | 1.01 | 1.08 |  | 1.06 | 1.03 |  | 1.02 | 1 | 1.19 | 1.05 |
|  | (0.98-1.06) |  | (0.97-1.04) | (1.02-1.14) |  | (1.02-1.11) | (0.99-1.06) |  | (0.96-1.08) | (0.96-1.04) | (1.10-1.28) | (1.00-1.11) |
| Overall CVH | 1.06 |  | 1.03 | 1.32 |  | 1.09 | 1.07 |  | 1.03 | 1.02 | 1.51 | 1.23 |
|  | (1.03-1.09) |  | (1.00-1.06) | (1.25-1.40) |  | (1.05-1.13) | (1.04-1.10) |  | (0.99-1.07) | (0.99-1.06) | (1.41-1.63) | (1.15-1.31) |
| ^*^ Ideal behavioral CVH was defined as having 3-4 out of 4 behavioral metrics (smoking, PA, diet, and BMI) at ideal status. Ideal biological CVH was defined as having 3 biological metrics (blood pressure, fasting plasma glucose, and total cholesterol) at ideal status. Ideal overall CVH was defined as having 5-7 out of 7  total metrics at ideal status  ^†^ Adjusted for age (continuous) and sex; ^‡^ Adjusted for age (continuous); ^§^ Adjusted for sex | | | | | | | | | | | | |

| **S7 Table.**  **Relative inequalities (RII, 95%CI) of achieving ideal CVH* by sex and age category 2013-2017** | | | | | | | | | | | | |
| --- | --- | --- | --- | --- | --- | --- | --- | --- | --- | --- | --- | --- |
|  | Total  population^†^ |  | By sex^‡^ | |  | By age category^§^ | |  | By sex and age category^‡^ | | | |
|  |  |  | Male | Female |  | 25-49 years | 50-64 years |  | 25-49 years | 50-64 years male | 25-49 years | 50-64 years |
|  |  |  |  |  |  |  |  |  | male | male | female | female |
| CVH metrics |  |  |  |  |  |  |  |  |  |  |  |  |
| Smoking | 1.25 |  | 1.20 | 3.68 |  | 1.3 | 1.13 |  | 1.22 | 1.11 | 3.7 | 4.08 |
|  | (1.21-1.30) |  | (1.15-1.24) | (2.99-4.50) |  | (1.24-1.37) | (1.07-1.19) |  | (1.17-1.29) | (1.05-1.18) | (2.99-4.59) | (1.97-8.42) |
| PA | 1.12 |  | 1.14 | 1.11 |  | 1.08 | 1.23 |  | 1.08 | 1.27 | 1.09 | 1.2 |
|  | (1.08-1.17) |  | (1.06-1.23) | (1.06-1.16) |  | (1.03-1.14) | (1.15-1.32) |  | (0.98-1.19) | (1.14-1.42) | (1.03-1.15) | (1.11-1.31) |
| Diet | 1.12 |  | 1.09 | 1.15 |  | 1.11 | 1.19 |  | 1.15 | 1.2 | 1.14 | 1.18 |
|  | (1.09-1.16) |  | (1.04-1.16) | (1.11-1.20) |  | (1.07-1.16) | (1.10-1.28) |  | (1.10-1.20) | (1.09-1.32) | (1.10-1.19) | (1.08-1.29) |
| BMI | 1.03 |  | 0.90 | 1.38 |  | 1.04 | 1.05 |  | 0.9 | 0.90 | 1.42 | 1.33 |
|  | (0.99-1.07) |  | (0.85-0.95) | (1.28-1.47) |  | (0.98-1.09) | (0.98-1.12) |  | (0.84-0.96) | (0.82-0.97) | (1.30-1.55) | (1.20-1.48) |
| BP | 1.13 |  | 1.07 | 1.33 |  | 1.14 | 1.16 |  | 1.02 | 1.12 | 1.68 | 1.22 |
|  | (1.07-1.20) |  | (1.00-1.14) | (1.22-1.46) |  | (1.05-1.24) | (1.08-1.24) |  | (0.92-1.12) | (1.03-1.22) | (1.44-1.97) | (1.00-1.36) |
| FPG | 1.34 |  | 1.19 | 1.83 |  | 1.44 | 1.26 |  | 1.16 | 1.21 | 2.29 | 1.44 |
|  | (1.23-1.46) |  | (1.07-1.32) | (1.59-2.11) |  | (1.26-1.63) | (1.13-1.41) |  | (0.99-1.37) | (1.06-1.38) | (1.86-2.82) | (1.19-1.74) |
| TC | 0.92 |  | 0.85 | 1.01 |  | 0.94 | 0.91 |  | 0.85 | 0.83 | 1.09 | 0.96 |
|  | (0.86-0.97) |  | (0.77-0.93) | (0.93-1.09) |  | (0.86-1.02) | (0.84-0.98) |  | (0.75-0.96) | (0.72-0.95) | (0.96-1.24) | (0.87-1.06) |
| CVH status |  |  |  |  |  |  |  |  |  |  |  |  |
| Behavioral CVH | 1.09 |  | 1.05 | 1.29 |  | 1.09 | 1.09 |  | 1.05 | 1.04 | 1.29 | 1.36 |
|  | (1.06-1.11) |  | (1.02-1.07) | (1.23-1.34) |  | (1.06-1.12) | (1.05-1.13) |  | (1.02-1.08) | (1.00-1.06) | (1.23-1.35) | (1.24-1.44) |
| Biological CVH | 1.02 |  | 1.01 | 1.03 |  | 1.07 | 1.03 |  | 1.02 | 1 | 1.21 | 1.06 |
|  | (0.96-1.09) |  | (0.97-1.05) | (0.97-1.09) |  | (1.02-1.12) | (0.99-1.06) |  | (0.97-1.08) | (0.96-1.05) | (1.11-1.31) | (1.01-1.11) |
| Overall CVH | 1.06 |  | 1.03 | 1.34 |  | 1.1 | 1.07 |  | 1.04 | 1.02 | 1.57 | 1.25 |
|  | (1.03-1.09) |  | (1.00-1.06) | (1.26-1.42) |  | (1.05-1.14) | (1.04-1.11) |  | (0.99-1.08) | (0.99-1.06) | (1.45-1.69) | (1.16-1.34) |

^*^ Ideal behavioral CVH was defined as having 3-4 out of 4 behavioral metrics (smoking, PA, diet, and BMI) at ideal status. Ideal biological CVH was defined as having 3 biological metrics (blood pressure, fasting plasma glucose, and total cholesterol) at ideal status. Ideal overall CVH was defined as having 5-7 out of 7

total metrics at ideal status

^†^ Adjusted for age (continuous) and sex; ^‡^ Adjusted for age (continuous); ^§^ Adjusted for sex

**S1 Figure. World Health Organization (WHO) Commission on Social Determinants of Health (CSDH) framework model**

**
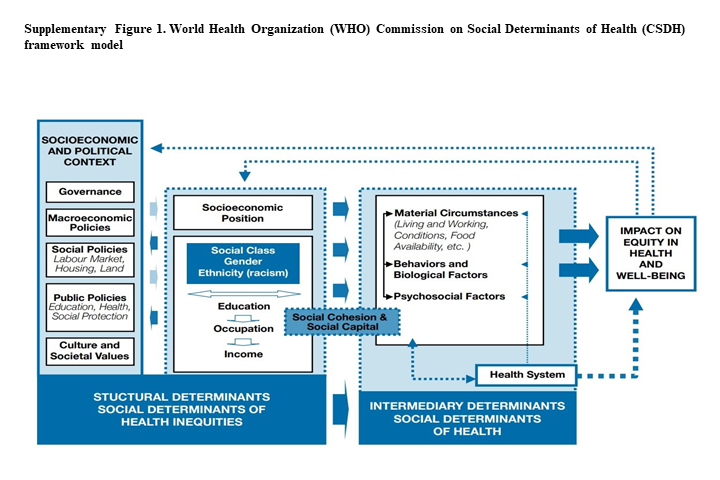
**

**S2 Figure. Flow chart of the participant enrollment in current study
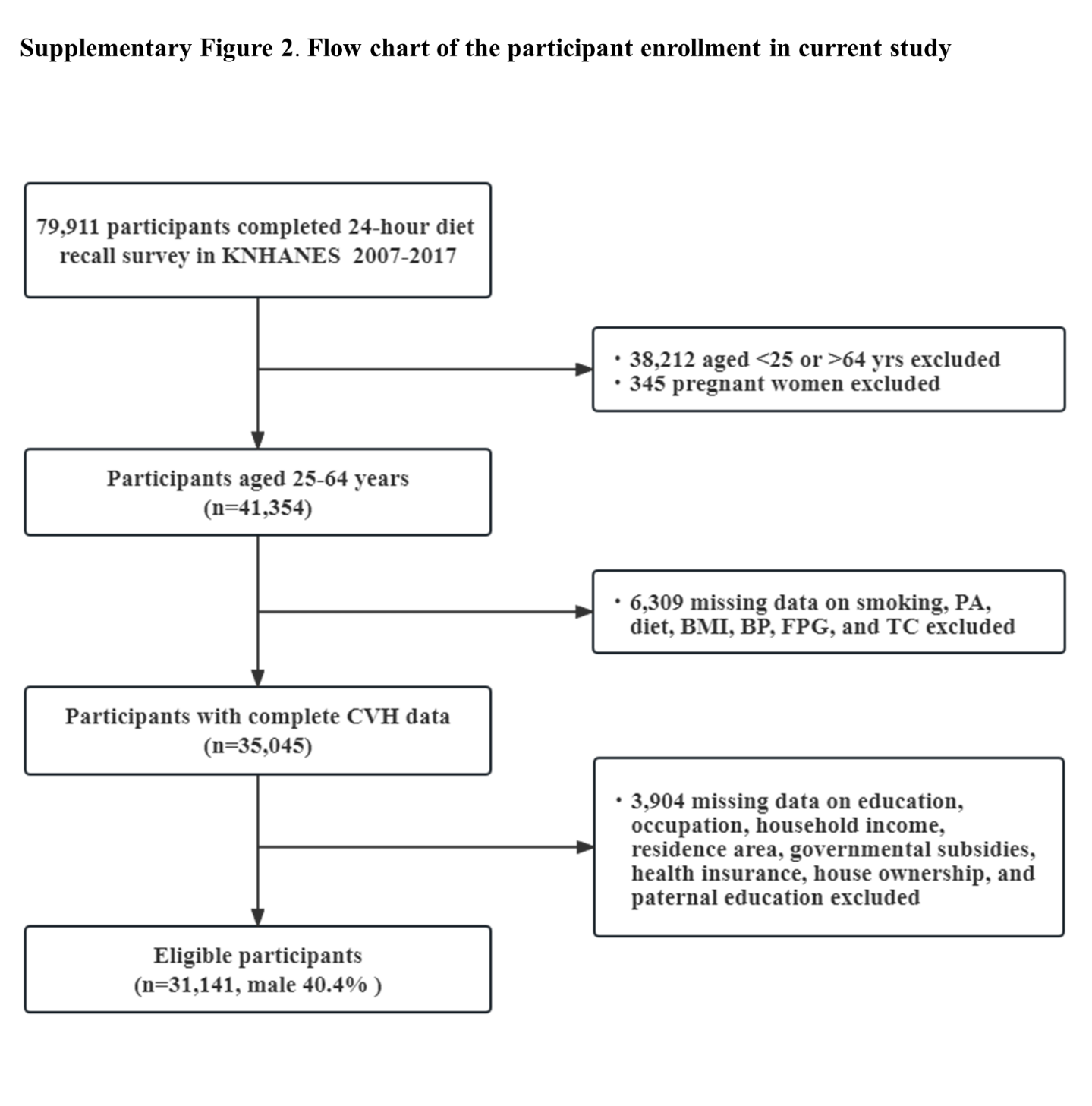
**

**S3 Figure. Multivariate-adjusted odds ratios (95%CI)* of ideal CVH**^†^  **by sex and educational attainment
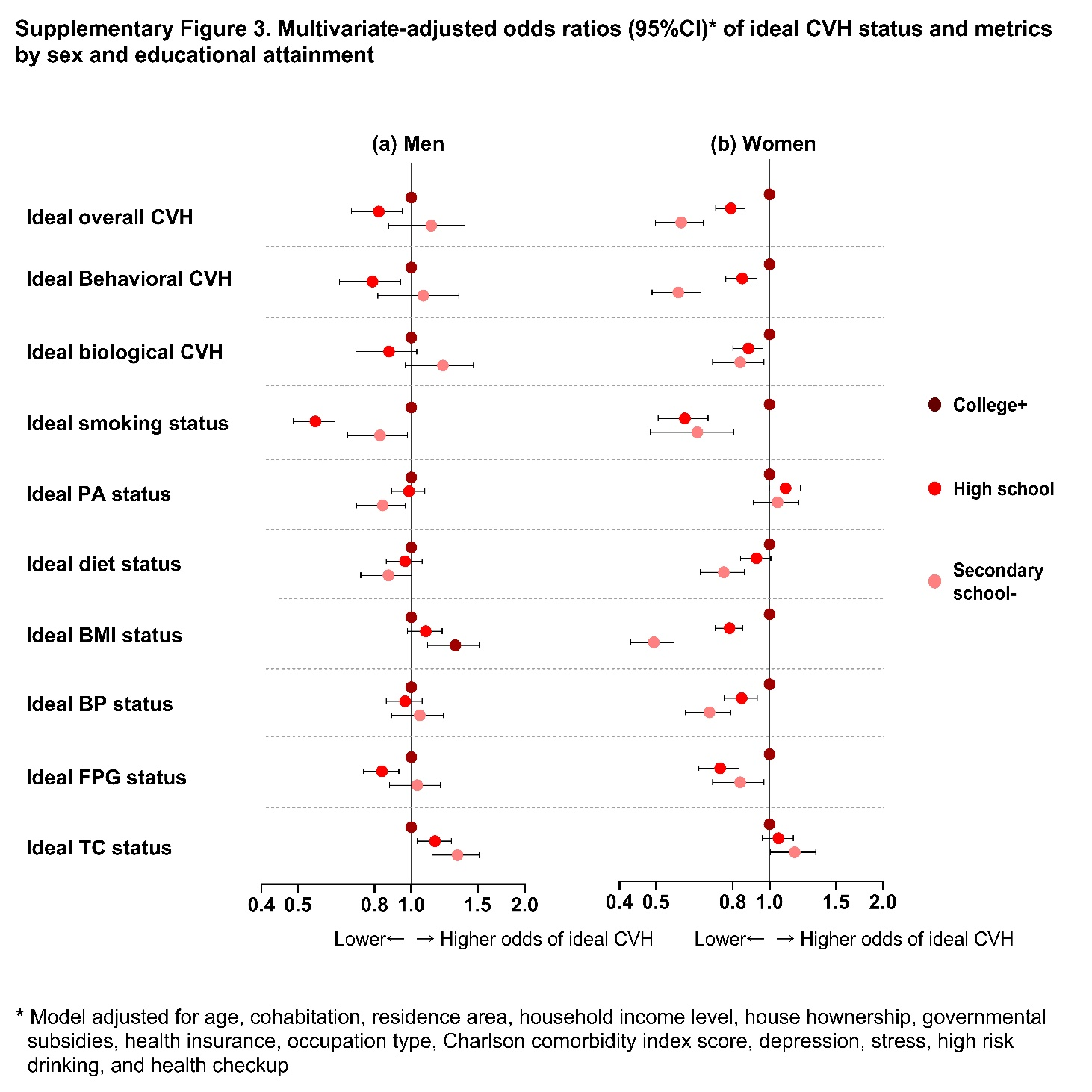
**

* Model adjusted for age, cohabitation, residence area, household income level, house ownership, governmental

subsidies, health insurance, occupation type, Charlson comorbidity index score, depression, stress, high risk

drinking, and health checkup

^†^ Ideal behavioral CVH was defined as having 3-4 out of 4 behavioral metrics (smoking, PA, diet, and BMI) at ideal

status. Ideal biological CVH was defined as having 3 biological metrics (blood pressure, fasting plasma glucose,

and total cholesterol) at ideal status. Ideal overall CVH was defined as having 5-7 out of 7 total metrics at ideal status

**S4 Figure. Multivariate-adjusted odds ratios (95%CI)* of ideal CVH**^†^ **by sex and household income level
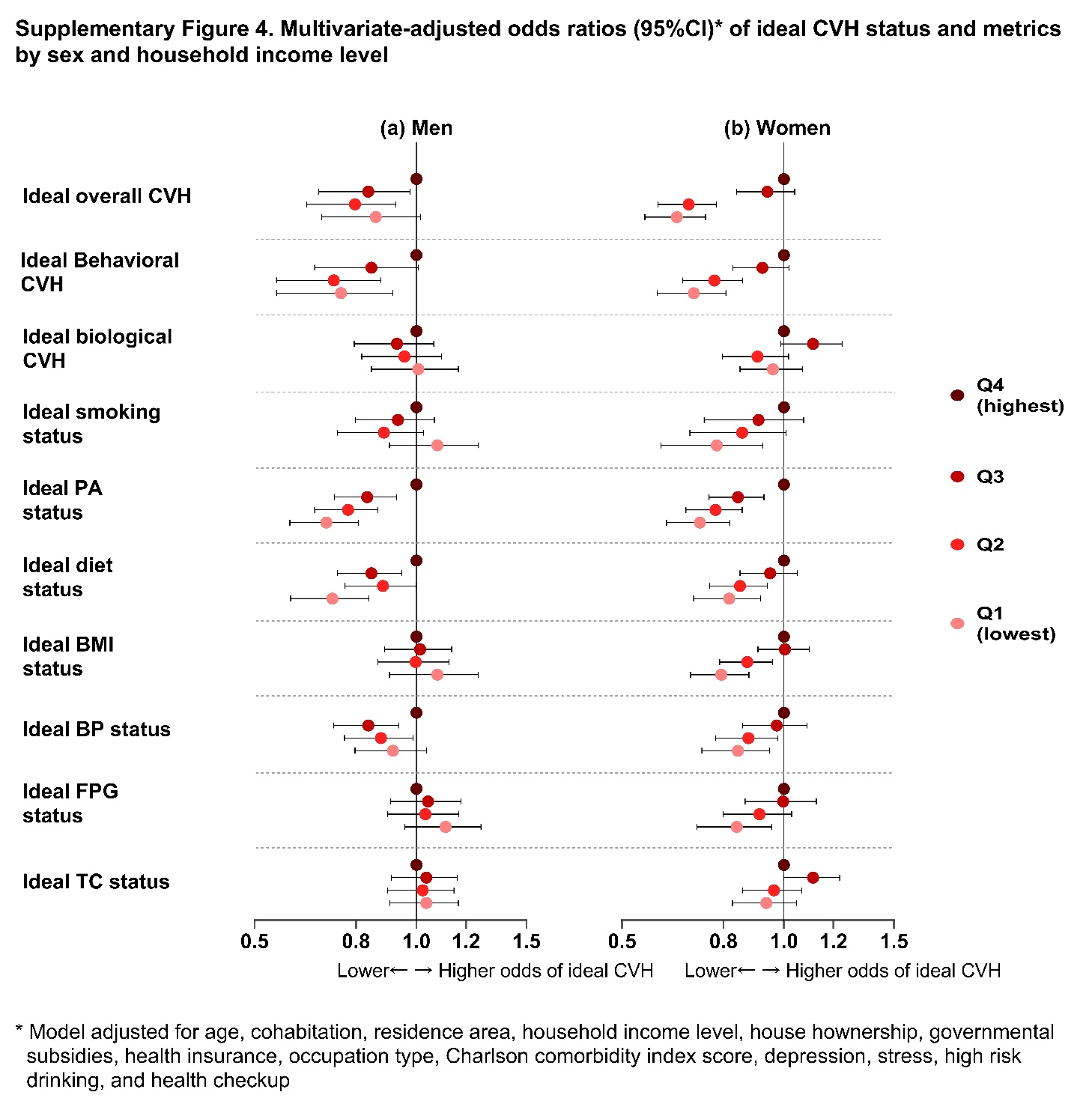
**

* Model adjusted for age, cohabitation, residence area, household income level, house ownership, governmental

subsidies, health insurance, occupation type, Charlson comorbidity index score, depression, stress, high risk

drinking, and health checkup

^†^ Ideal behavioral CVH was defined as having 3-4 out of 4 behavioral metrics (smoking, PA, diet, and BMI) at ideal

status. Ideal biological CVH was defined as having 3 biological metrics (blood pressure, fasting plasma glucose,

and total cholesterol) at ideal status. Ideal overall CVH was defined as having 5-7 out of 7 total metrics at ideal status

**S5 Figure. Age-standardized prevalence* of ideal CVH**^†^ **by sex and SES 2007-2012**

**
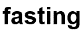

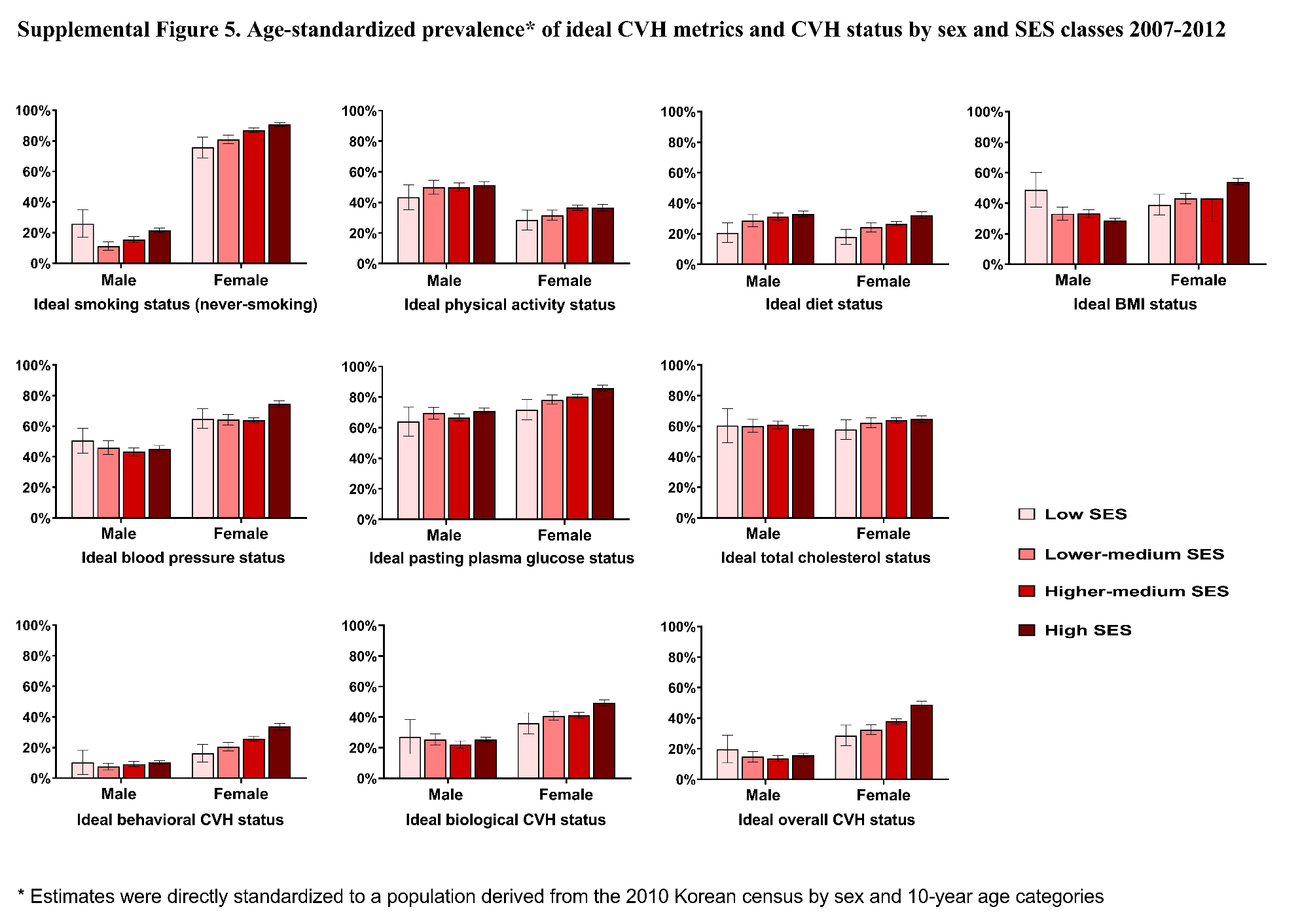
**

* Estimates were directly standardized to a population derived from the 2010 Korean census by sex and 10-year age categories

^†^ Ideal behavioral CVH was defined as having 3-4 out of 4 behavioral metrics (smoking, PA, diet, and BMI) at ideal status. Ideal biological CVH

was defined as having 3 biological metrics (blood pressure, fasting plasma glucose, and total cholesterol) at ideal status. Ideal overall CVH was

defined as having 5-7 out of 7 total metrics at ideal status

**S6 Figure. Age-standardized prevalence* of ideal CVH by sex and SES 2013-2017**

**
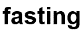

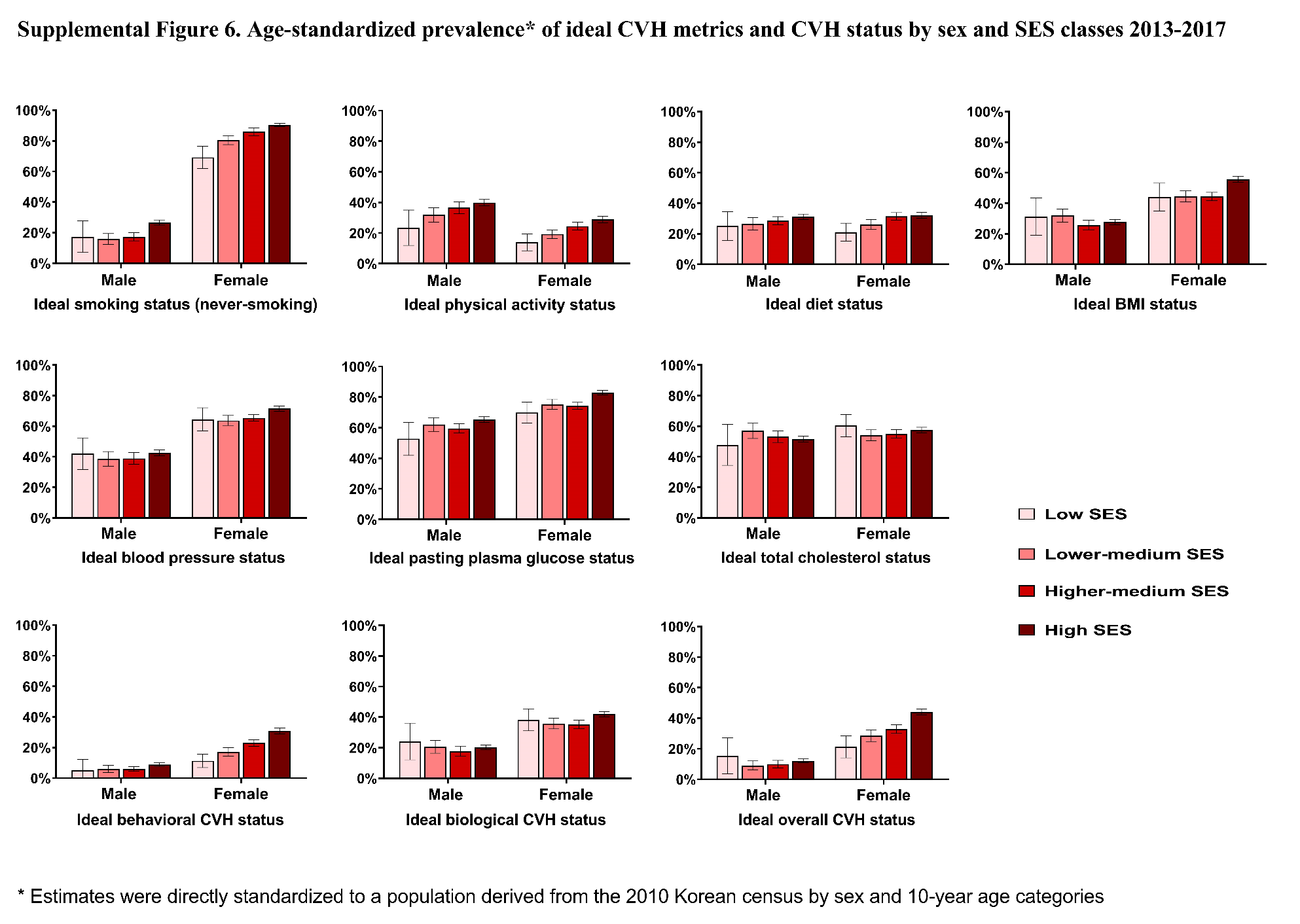
**

* Estimates were directly standardized to a population derived from the 2010 Korean census by sex and 10-year age categories

^†^ Ideal behavioral CVH was defined as having 3-4 out of 4 behavioral metrics (smoking, PA, diet, and BMI) at ideal status. Ideal biological CVH

was defined as having 3 biological metrics (blood pressure, fasting plasma glucose, and total cholesterol) at ideal status. Ideal overall CVH was

defined as having 5-7 out of 7 total metrics at ideal status
